# β-arrestin-2 regulates NMDA receptor function in spinal lamina II neurons and duration of persistent pain

**DOI:** 10.1038/ncomms12531

**Published:** 2016-08-19

**Authors:** Gang Chen, Rou-Gang Xie, Yong-Jing Gao, Zhen-Zhong Xu, Lin-Xia Zhao, Sangsu Bang, Temugin Berta, Chul-Kyu Park, Mark Lay, Wei Chen, Ru-Rong Ji

**Affiliations:** 1 Department of Anesthesiology, Duke University Medical Center, Durham, North Carolina 27710, USA; 2 Jiangsu Key Laboratory of Neuroregeneration, Co-Innovation Center of Neuroregeneration, Nantong University, Nantong, Jiangsu 226001, China; 3 Department of Anesthesiology and Pain Management, Xijing Hospital, Department of Neuroscience, Fourth Military Medical University, Xian, Shanxi 710032, China; 4 Pain Research Laboratory, Institute of Nautical Medicine, Co-Innovation Center of Neuroregeneration, Nantong University, Nantong, Jiangsu 226001, China; 5 Department of Neurobiology, Institute of Neuroscience, Key Laboratory of Medical Neurobiology of the Ministry of Health of China, Zhejiang University School of Medicine, Hangzhou, Zhejiang 3100058, China; 6 Pain Research Center, Department of Anesthesiology, University of Cincinnati Medical Center, Cincinnati, Ohio 45267, USA; 7 Department of Physiology, College of Medicine, Gachon University, Incheon 21999, South Korea; 8 Department of Medicine, Duke University Medical Center, Durham, North Carolina 27710, USA; 9 Department of Neurobiology, Duke University Medical Center, Durham, North Carolina 27710, USA

## Abstract

Mechanisms of acute pain transition to chronic pain are not fully understood. Here we demonstrate an active role of β-arrestin 2 (Arrb2) in regulating spinal cord NMDA receptor (NMDAR) function and the duration of pain. Intrathecal injection of the mu-opioid receptor agonist [D-Ala^2^, NMe-Phe^4^, Gly-ol^5^]-enkephalin produces paradoxical behavioural responses: early-phase analgesia and late-phase mechanical allodynia which requires NMDAR; both phases are prolonged in *Arrb2* knockout (KO) mice. Spinal administration of NMDA induces GluN2B-dependent mechanical allodynia, which is prolonged in *Arrb2*-KO mice and conditional KO mice lacking *Arrb2* in presynaptic terminals expressing Nav1.8. Loss of *Arrb2* also results in prolongation of inflammatory pain and neuropathic pain and enhancement of GluN2B-mediated NMDA currents in spinal lamina IIo not lamina I neurons. Finally, spinal over-expression of *Arrb2* reverses chronic neuropathic pain after nerve injury. Thus, spinal Arrb2 may serve as an intracellular gate for acute to chronic pain transition via desensitization of NMDAR.

## Abstract (web summary)

The cellular mechanisms underlying acute pain transitions to chronic pain are poorly understood. Here the authors show that the scaffolding protein β-arrestin 2 contributes to these processes via desensitization of NMDA receptors in spinal neurons.

## Graphical abstract

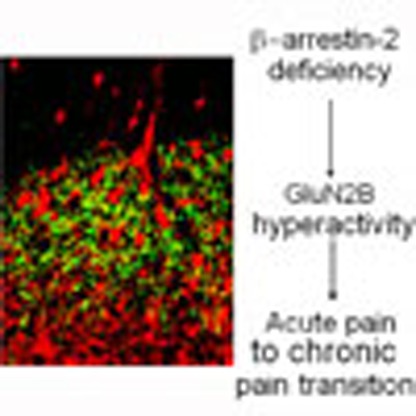

The past decade has seen considerable progress in revealing how inflammatory and neuropathic pain is induced by inflammatory mediators via sensitization of primary sensory neurons (peripheral sensitization)^1^,^2^ and subsequent sensitization of spinal cord neurons (central sensitization)^3^,^4^,^5^. In particular, activation of NMDA receptors (NMDAR) in spinal cord dorsal horn (SDH) nociceptive neurons plays an essential role in driving hypersensitivity in spinal cord pain circuit^3^,^6^,^7^. However, little is known about how acute pain naturally resolves. Disruption of the active resolution processing may result in transition from acute pain to chronic pain^8^. Activation of endogenous G protein-coupled receptors (GPCR) such as opioid receptors in SDH was implicated in the resolution of inflammatory pain^9^. Inflammatory pain is also prolonged in mice lacking GPCR kinase 2 (GRK2) in primary sensory neurons^10^.

GPCRs are desensitized by GRKs via receptor phosphorylation and subsequent binding of β-arrestin to the phosphorylated GPCRs^11^. β-arrestin are multifunctional scaffold proteins that regulate receptor endocytosis, signalling, trafficking and ubiquitination^12^. β-arrestin-1 (Arrb1) and β-arrestin-2 (Arrb2) are two of the major Arrestin family members and play different roles in GPCR desensitization and signalling^11^. Arrb2 was implicated in μ-opioid receptor (MOR) desensitization, and mice lacking *Arrb2* exhibited enhanced and prolonged morphine analgesia^13^. Similarly, intrathecal (i.t.) pretreatment with Arrb2 antibody potentiated the antinociception induced by i.t. MOR agonist [D-Ala^2^, NMe-Phe^4^, Gly-ol^5^]-enkephalin (DAMGO)^14^. However, antinociceptive tolerance, which is critically dependent on spinal NMDAR^15^, still developed in *Arrb2* knockout (KO) mice in some pain test^16^. It was also suggested that MOR desensitization by Arrb2 determines morphine tolerance but not dependence^17^. It was further suggested that MOR activity is differentially regulated in diverse regions of the central nervous system (spinal cord versus brain) in *Arrb2* KO mice^16^. In addition to GPCRs, Arrb2 modulates the surface molecules TGF-β receptors^18^ and Wnt5A-receptor^19^ and intracellular signalling molecules such as MAP kinases^12^. Arrb1 and Arrb2 also regulate the transient receptor potential (TRP) ion channels, such as ubiquitination of TRPV4 by Arrb1 (ref. 20) and desensitization of TRPV1 by Arrb2 (ref. 21). Despite these molecular mechanisms of Arrb2, the overall roles of Arrb2 in regulating NMDAR function and inflammatory and neuropathic pain are unclear. In this study, we demonstrate that Arrb2 contributes to the transition of acute to chronic pain and also the resolution of chronic neuropathic pain via desensitization of NMDAR in SDH lamina II neurons.

## Results

### Opioid-induced LTP is abolished after Arrb2 deficiency

MOR agonists, such as morphine remain to be the first-line treatment for severe pain. However, MOR agonist not only induces acute analgesia but also causes paradoxical hyperalgesia^22^. In particular, opioid withdrawal induces NMDAR-dependent long-term potentiation (LTP) in spinal cord pain circuit, which may underlie opioid-induced hyperalgesia^23^. We set out to assess if Arrb2 plays different roles in opioid-induced LTP, analgesia and hyperalgesia/allodynia using a specific MOR agonist DAMGO.

**Figure 1:**
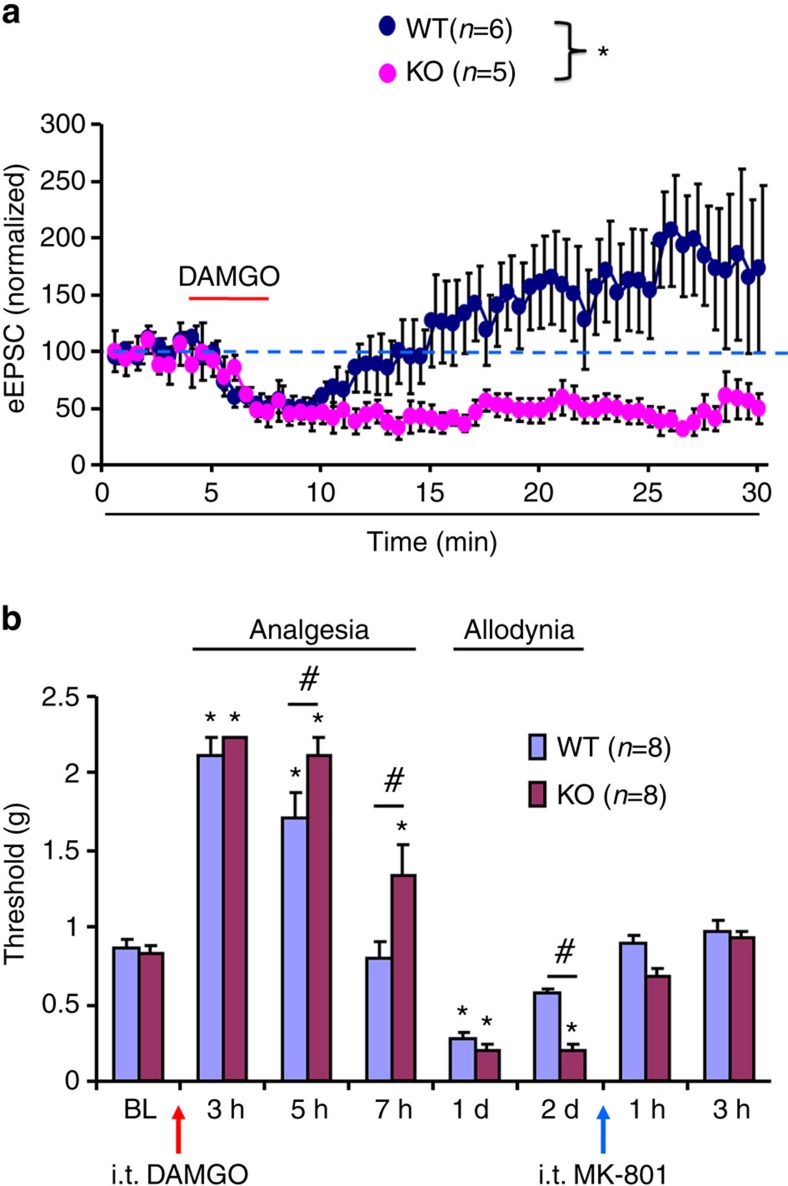
*Arrb2* regulates sLTP and DAMGO-induced analgesia and allodynia. (**a**) Normalized amplitudes of dorsal root stimulation evoked EPSCs (eEPSCs) in lamina IIo neurons before, during and after DAMGO (500 nM) perfusion in spinal cord slices in wild-type (WT) and *Arrb2***-**knockout (KO) mice. In WT mice, DAMGO produces initial suppression of EPSCs followed by long-term potentiation (LTP) of eEPSCs during withdrawal phase. Spinal LTP (sLTP) is abolished in KO mice, only showing prolonged suppression of eEPSCs. *P<0.05, Two-Way ANOVA (WT. versus KO), *n*=5, 6 neurons per group. (**b**) von Frey test shows acute analgesia and late-onset mechanical allodynia following i.t. DAMGO (1 μg) in WT and KO mice and the reversal of allodynia by MK-801 (i.t., 10 nmol). *P<0.05, versus baseline (BL), ^#^*P*<0.05 (WT versus KO). Two-Way ANOVA, *n*=8 mice per group. All the data are mean±s.e.m. d, day.

First, we compared spinal cord LTP induction in wild-type (WT) and *Arrb2*-KO mice. As previously reported^23^, incubation of spinal cord slices with 0.5 μM DAMGO induced rapid suppression of dorsal root stimulation-evoked excitatory postsynaptic currents (eEPSCs) in lamina IIo neurons. These interneurons form a nociceptive circuit by receiving input from C-fibre afferents and sending output to lamina I projection neurons^24^,^25^,^26^,^27^. Also as previously reported^23^, the DAMGO-induced short-term depression of eEPSC’s was followed by LTP of eEPSCs after DAMGO withdrawal (Fig. 1a). Interestingly, DAMGO withdrawal-evoked LTP was abolished by deletion of *Arrb2*, and *Arrb2*-KO mice exhibited persistent suppression of eEPSCs (Fig. 1a). This result indicates an enhancement of spinal MOR activity in *Arrb2*-KO mice, in support of a previous study showing increased binding of MOR in brainstem of KO mice^17^.

### Arrb2 regulates opioid-induced analgesia and allodynia

Next, we tested whether opioid-induced analgesia is altered in *Arrb2*-KO mice. It was shown in hot plate test that systemic DAMGO-induced analgesia was potentiated and prolonged in KO mice^13^. Opioid is also known to cause hyperalgesia^22^. To define the spinal cord mechanisms of Arrb2 in opioid-induced analgesia and hyperalgesia/allodynia, we also tested mechanical pain sensitivity after i.t. DAMGO (1 μg) in WT and KO mice using von Frey hairs. Interestingly, i.t. DAMGO induced time-dependent changes of mechanical sensitivity in WT mice: hypoalgesia (analgesia) in early-phase (0.5–5 h) followed by mechanical allodynia in late-phase (24–48 h). Notably, the duration of both DAMGO-induced acute analgesia and persistent mechanical allodynia was prolonged in KO mice (Fig. 1b). At 7 h after the DAMGO injection, WT but not KO mice showed a full recovery of analgesia (Fig. 1b). Compared with WT mice, DAMGO-induced analgesia was also enhanced in KO mice at 5 h (Fig. 1b). DAMGO produced paradoxical allodynia at 24 h in both WT and KO mice (Fig. 1b). However, mechanical allodynia after i.t. DAMGO was prolonged in KO mice and recovered in 2 days and 4 days in WT and KO mice, respectively (Supplementary Fig. 1a).

Given a critical role of NMDAR in spinal cord synaptic plasticity (central sensitization)^8^ and opioid-induced hyperalgesia^15^, we tested the involvement of NMDAR in opioid-induced mechanical allodynia in WT and *Arrb2*-KO mice. As expected, spinal administration of the NMDAR antagonist MK-801 (i.t., 10 nmol) reversed DAMGO-induced mechanical allodynia in WT and KO mice (Fig. 1b; Supplementary Fig. 1b). We also measured the activity of NMDAR in SDH by recording NMDA (50 μM)-induced inward currents in lamina IIo neurons of spinal cord slices in mice 24 h after DAMGO treatment. In agreement with the behavioural finding, NMDA-induced currents in lamina IIo neurons were also enhanced in DAMGO-treated WT mice at 24 h (Supplementary Fig. 1c,d).

Altogether, these results suggest that Arrb2 not only contributes to opioid-induced acute analgesia as previously shown^13^, but also contributes to opioid-induced late-phase allodynia, and the latter is mediated by spinal NMDAR.

### NMDA-induced allodynia is prolonged in mice lacking *Arrb2*

**Figure 2:**
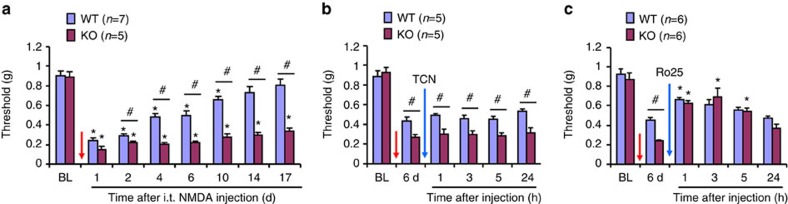
*Arrb2* deficiency causes prolongation of NMDA-induced allodynia. (**a**) Prolongation of NMDA (1 nmol, i.t.)-induced mechanical allodynia in *Arrb2* KO mice. *P<0.05, versus baseline (BL), ^#^*P*<0.05 (KO versus WT), Two-Way ANOVA, *n*=5, 7 mice per group. (**b**) GluN2A antagonist TCN-201 (10 nmol, i.t.) fails to reduce the NMDA-induced allodynia. (**c**) GluN2B antagonist Ro25-6091 (10 nmol, i.t.) reduces the NMDA-induced allodynia in WT and KO mice. *P<0.05, versus pre-NMDA injection baseline (6 d), One-Way ANOVA, *n*=6 mice per group. ^#^*P*<0.05 (WT versus KO), Two-Way ANOVA, *n*=6 mice per group. BL, baseline. The antagonists were given 6 d after i.t. NMDA injection. Note that the difference between WT and KO mice is abolished by the GluN2B inhibition. All the data are mean±s.e.m. d, day.

Next, we employed pharmacological approaches to test the function of spinal NMDARs in WT and *Arrb2*-KO mice. Spinal NMDA injection (i.t., 1 nmol) elicited persistent mechanical allodynia in WT mice, which resolved at 10 days (Fig. 2a). In contrast, NMDA-evoked allodynia was prolonged in KO mice, showing no sign of recovery at 17 days (Fig. 2a). Thus, a single i.t. NMDA could elicit chronic pain after *Arrb2* deletion. Functional assembly of NMDAR requires both NR1 (GluN1) and NR2A (GluN2A) or NR2B (GluN2B) subunits, and GluN2A and GluN2B play different roles in synaptic plasticity and chronic pain^3^,^7^,^28^. NMDA-induced persistent mechanical allodynia was not affected by GluN2A antagonist TCN (i.t., 10 nmol, Fig. 2b). However, i.t. GluN2B antagonist Ro25 (10 nmol) reduced this allodynia in both WT and KO mice (Fig. 2b,c), suggesting that spinal GluN2B plays a predominant role in i.t. NMDA-induced persistent pain.

### Arrb2 regulates synaptic GluN2B in SDH lamina IIo neurons

**Figure 3:**
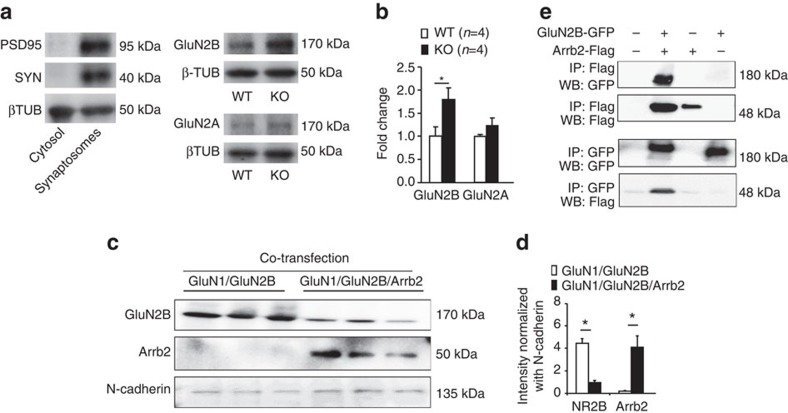
*Arrb2* deficiency increases synaptic GluN2B in SDH neurons. (**a**,**b**) Expression of GluN2A and GluN2B in synaptosome-containing P2 fraction of spinal cord dorsal horn (SDH) of WT and *Arrb2*-KO mice. (**a**) Enrichment of synaptic proteins PSD-95 and synaptophysin (SYN) in synaptosome-contacting P2 fraction but not cytosol fraction. (**b**) Quantification of GluN2A and GluN2B expression. *P<0.05, Student *t*-test, *n*=4 mice per group. (**c**) Surface expression of GluN2B and Arrb2 in HeLa cells transfected with GluN1/GluN2B and GluN1/GluN2B/Arrb2. (**d**) Relative expression levels of GluN2B and Arrb2, normalized with N-cadherin, a positive control for surface expression. *P<0.05, Student’s *t*-test, *n*=3 cultures per group. (**e**) Pull down assay showing Co-IP of Arrb2 with GluN2B in HeLa cells. All the data are mean±s.e.m. Gel images have been cropped for presentation. Full size images are presented in Supplementary Fig. 8a.

Given an important role of Arrb2 in regulating the trafficking and surface expression of GPCRs^11^, we reasoned that Arrb2 might also modulate synaptic and surface expression of NMDARs in SDH. We prepared synaptosomes (P2 preparation containing synaptosomes)^29^ from SDHs and compared the expression of GluN2A and GluN2B. As expected, the synaptic proteins PSD95 and synaptophysin were highly enriched in this synaptosome-like preparation (Fig. 3a). Of interest the expression of GluN2B but not GluN2A was increased in KO mice (Fig. 3a,b). Biotinylation experiment showed that the surface expression of Arrb2 was inversely correlated with that of GluN2B in Hela cells: high expression of Arrb2 was associated with low expression of GluN2B, and *vice versa* (Fig. 3c,d). However, Arrb2 failed to regulate GluN2A surface expression in Hela cells (Supplementary Fig. 2a,b). Pull down analysis showed co-IP of Arrb2 with GluN2B (Fig. 2d) not GluN2A (Supplementary Fig. 2c) in Hela cells, suggesting a specific interaction between Arrb2 and GluN2B.

**Figure 4:**
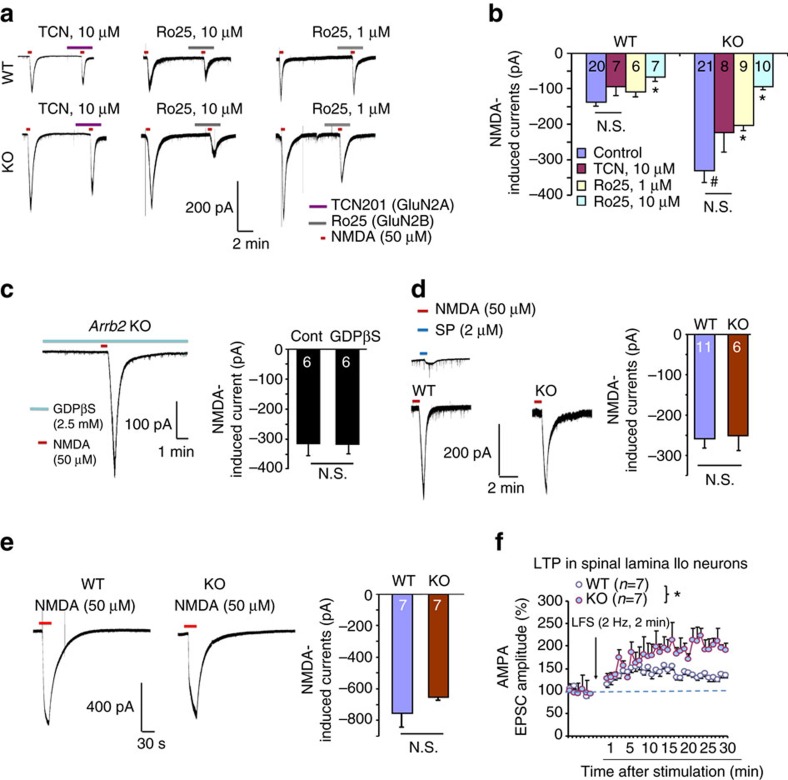
*Arrb2* deficiency enhances GluN2B currents in spinal lamina IIo neurons. (**a**) Representative traces of inward currents in WT and KO mice, induced by NMDA (50 μM) via bath application. Note a remarkable potentiation of the current in KO mice. Also note different effects of TCN-201 (GluN2A antagonist) and Ro25-6091 (GluN2B antagonist). (**b**) Amplitude of NMDA-induced currents from (a). *P<0.05, versus corresponding control, ^#^*P*<0.05 (WT versus KO), One-Way ANOVA, *n*=6–21 neurons per group (shown in each column). N.S., not significant. (**c**) Representative trace of NMDA current in KO mice in the presence of G-protein inhibitor GDPβS (2.5 mM) via intracellular delivery through the recording pipette. Right, amplitude of NMDA-induced currents. N.S., no significance, Student’s *t*-test, *n*=6 neurons per group. (**d,e**) NMDA currents in spinal lamina I neurons and hippocampal CA1 neurons are comparable in WT and *Arrb2*-KO mice. (**d**) Traces of NMDA (50 μM)-induced currents in lamina I neurons of spinal slices. The projection neurons respond to substance P (2 μM). Right, amplitude of NMDA-induced currents. N.S., no significance, *n*=6 and 11 neurons per group. (**e**) Traces of NMDA (50 μM)-induced currents in hippocampal CA1 neurons from WT and KO mice. Right, amplitude of NMDA currents in hippocampal CA1 neurons. N.S., no significance, Student’s *t*-test, *n*=7 neurons per group. (**f**) Spinal LTP of C-fibre evoked EPSCs (eEPSCs) in lamina IIo neurons of spinal cord slices in WT and KO mice following low frequency dorsal root stimulation (LFS, 2 Hz). *P<0.05, WT versus KO, Two-way ANOVA, *n*=7 neurons per group. All data are expressed as mean±s.e.m.

To further determine the regulation of GluN2A and GluN2B function by Arrb2, we recorded total NMDA currents in lamina IIo neurons in spinal cord slices. Strikingly, NMDA (50 μM) induced current was dramatically increased by almost 2-fold, from 123.8±7.0 pA in WT mice to 313.5±39.5 pA in KO mice (Fig. 4a,b). Interestingly, intracellular G-protein inhibitor GDPβS had no effects on NMDA currents in Arrb2-deficient neurons (Fig. 4c). As positive controls, GDPβS inhibited GPCR signalling triggered by GABA_A_ agonist GABA and group I metabotropic glutamate receptor agonist DHPG (Supplementary Fig. 3a,b). Thus, Arrb2 deficiency results in an enhancement of total NMDA currents in lamina IIo neurons, in a G-protein-independent manner.

We also assessed the distinct contribution of GluN2A and GluN2B subunits to total NMDA currents in WT and KO mice. Compared with NMDA currents in WT mice, GluN2B antagonist Ro25 at 10 μM produced a greater inhibition of the currents in KO mice (70% inhibition in KO mice versus 45% inhibition in WT mice Fig. 4a,b). In contrast, GluN2A antagonist TCN (10 μM) had no significant inhibition of NMDA currents in WT and KO mice (Fig. 4a,b). For comparison, we also recorded total NMDA currents in spinal cord lamina I projection neurons and hippocampal CA1 neurons in WT and KO mice. Interestingly, we found that NMDA-induced currents in WT and KO mice were comparable in SDH lamina I projection neurons (Fig. 4d) and hippocampal CA1 neurons (Fig. 4e). NMDAR-dependent LTP is an important form of spinal cord synaptic plasticity underlying the genesis of chronic pain^30^. Low frequency stimulation (LFS, 2 Hz) of dorsal root C-fibre primary afferents was shown to elicit NMDAR-dependent spinal LTP (sLTP)^31^,^32^. Notably, this sLTP in lamina IIo neurons was greatly potentiated in KO mice (Fig. 4f). Collectively, these data indicate that (1) Arrb2 is a negative regulator of spinal NMDAR and (2) this regulation is GluN2B-dependent and also region-specific.

### Arrb2 is expressed in neurons and axonal terminals in SDH

**Figure 5:**
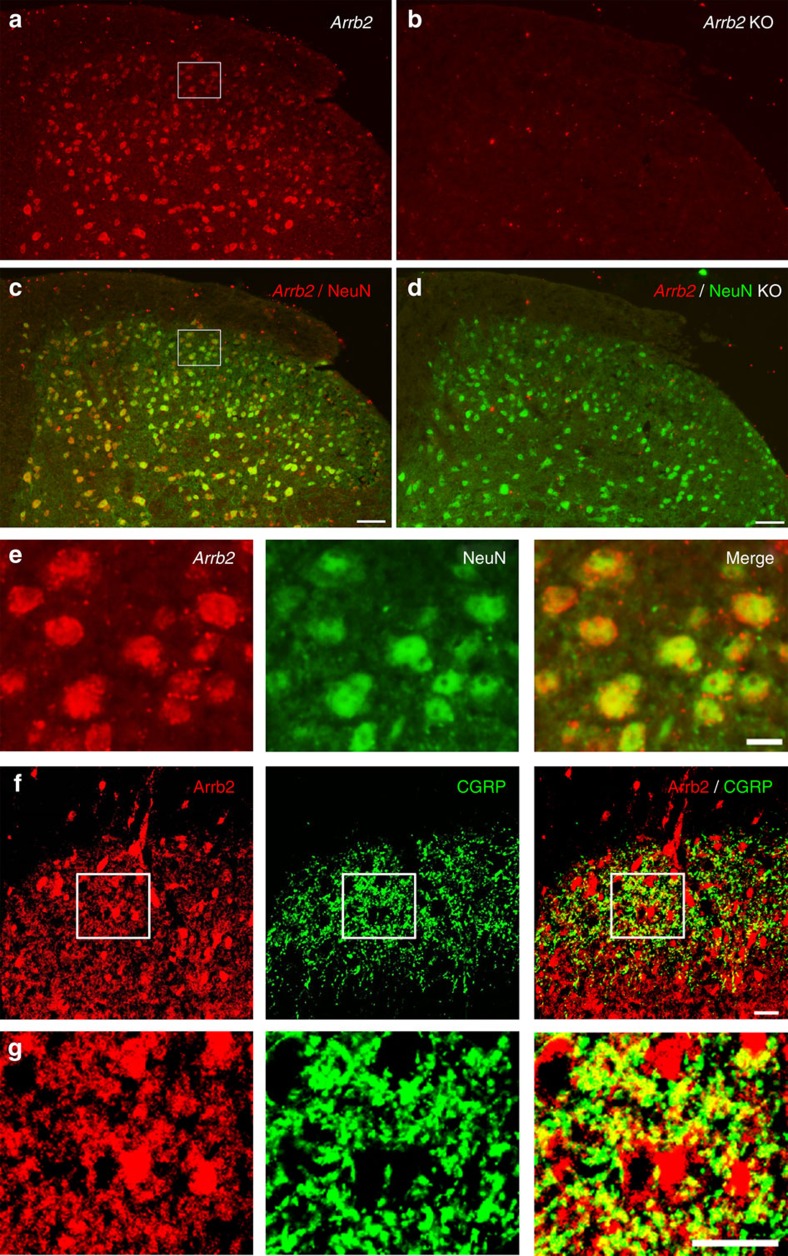
Expression of *Arrb2* mRNA and Arrb2 protein in SDH. (**a-d**) Double staining of *in situ* hybridization and immunohistochemistry showing colocalization of *Arrb2* mRNA and NeuN immunoreactivity in SDH neurons. (**a**,**c**) Colocalization of *Arrb2* mRNA and NeuN immunoreactivity in SDH neurons of WT mice. (**b**,**d**) Loss of *Arrb2* mRNA labelling in *Arrb2* KO mice. Scale, 50 μm. (**e**) Enlarged images from the boxes in (**a**) and (**c**) in the superficial SDH. Note that *Arrb2* mRNA is highly co-localized with NeuN in SDH neurons. Scale, 10 μm. (**f**) Double staining of Arrb2 and CGRP in axonal terminals in the superficial SDH. Scale, 20 μm. (**g**) High magnification images of boxes in f showing co-localization of Arrb2 and CGRP in axonal terminals but not in cell bodies. Scale, 20 μm.

Although Arrb2 is known to be expressed in the SDH(ref. 33), the expression pattern is not well characterized. *In situ* hybridization revealed that *Arrb2* mRNA is widely expressed in SDH of WT mice, although the staining is stronger in the deep dorsal horn (laminae III–VI, Fig. 5a). This staining was absent in *Arrb2*-KO mice (Fig. 5b), confirming the specificity of the *Arrb2* mRNA staining. Double staining of *in situ* hybridization (*Arrb2*) and immunohistochemistry (NeuN, a neuronal marker) showed that *Arrb2* is almost completely co-localized with NeuN in both the deep laminae (III–VI) and the superficial laminae I–II of SDH (Fig. 5c,e). This result indicates that majority of SDH neurons express *Arrb2* mRNA.

We also examined Arrb2 protein expression in spinal cord and dorsal root ganglia (DRG) using immunohistochemistry. We observed strong Arrb2 immunoreactivity all over the SDH (Fig. 5f,g). Arrb2 is also broadly expressed in DRG primary sensory neurons, and some of these Arrb2-postive neurons co-expressed calcitonin gene-related peptide (CGRP), a marker for peptidergic nociceptors (Supplementary Fig. 4a,b). In SDH CGRP is derived from primary afferents and, therefore can serve as a presynaptic marker^34^. Double staining shows that Arrb2 and CRGP are highly colocalized in superficial SDH (Fig. 5f,g). Therefore, Arrb2 is present at both presynaptic sites (central terminals of primary afferents co-expressing CGRP) and postsynaptic site (neuronal cell bodies) in SDH.

### Arrb2 controls inflammatory and neuropathic pain duration

**Figure 6:**
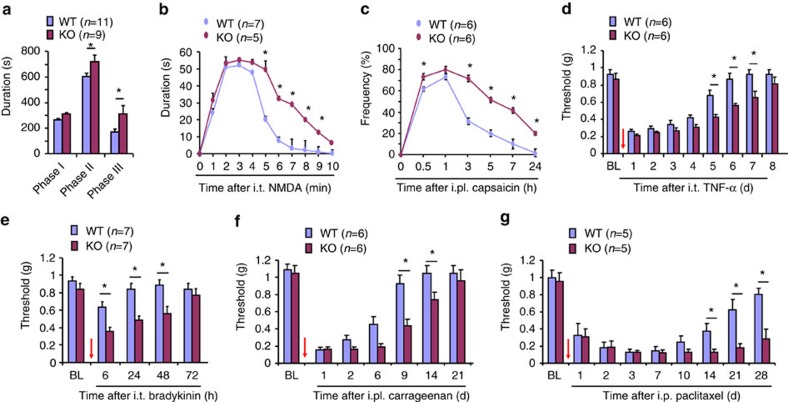
*Arrb2* deficiency causes prolongation of inflammatory and neuropathic pain. (**a-g**) Inflammatory pain (**a-f**) and neuropathic pain (**g**) in wildtype (WT) and *Arrb2*-knockout (KO) mice. (**a**) Acute inflammatory pain in Phase-I (0–10 min), Phase-II (10–45 min) and Phase-III (45–90 min) following intraplantar (i.pl.) formalin. (**b**) Spontaneous pain after i.t. NMDA (1 nmol). (**c**) 2nd mechanical allodynia following i.pl. capsaicin (5 μg), measured by frequency response to a 0.16 g filament. (**d**,**e**) Mechanical allodynia following i.t. TNF-α (20 ng, **d**) and i.t. bradykinin (1 μg, **e**). (**f**) Persistent inflammatory pain (mechanical allodynia) following i.pl. carrageenan (1.5%). (**g**) Neuropathic pain (mechanical allodynia) after intraperitoneal paclitaxel (6 mg/kg). *P<0.05 (WT versus KO), *n*=5–11 mice per group, Two-Way ANOVA followed by *post-hoc* Bonferroni test. Arrows (**d**-**g**) indicate drug injections. All data are expressed as mean±s.e.m.

Next, we assessed whether Arrb2 has an active role in regulating the duration of inflammatory and neuropathic pain, as these pains require the activation of spinal NMDAR^3^,^7^. We tested following types of inflammatory pains in WT and *Arrb2*-KO mice: the intraplantar formalin-induced spontaneous pain (the 2nd and 3rd phases are mediated by spinal cord mechanism, Fig. 6a), the i.t. NMDA-induced spontaneous pain (Fig. 6b), the intraplantar capsaicin-evoked 2nd mechanical allodynia (Fig. 6c), the i.t. TNF-α-evoked mechanical allodynia (Fig. 6d), and the i.t. bradykinin-evoked mechanical allodynia (Fig. 6e). All these centrally mediated inflammatory pains, via the activation of either GPCR (bradykinin receptors) or non-GPCR (TNF receptors and NMDAR), were potentiated and prolonged in KO mice (Fig. 6a–e; Supplementary Fig. 5a). Mechanical allodynia after i.t. bradykinin in KO mice was further prevented by the NMDAR blockade with MK-801 (Supplementary Fig 5a). We also induced persistent inflammatory pain via intraplantar carrageenan injection (1.5%) and persistent neuropathic pain via peritoneal paclitaxel injection (6 mg/kg, i.p.). Both carrageenan-induced inflammatory pain (mechanical allodynia and heat hyperalgesia) and paclitaxel-induced neuropathic pain (mechanical allodynia and cold allodynia) were prolonged in KO mice (Fig. 3f,g; Supplementary Fig. 5b,c). Thus, Arrb2 is required for regulating the duration and the resolution of inflammatory and neuropathic pain.

### Presynaptic Arrb2 regulates NMDA currents and pain

**Figure 7:**
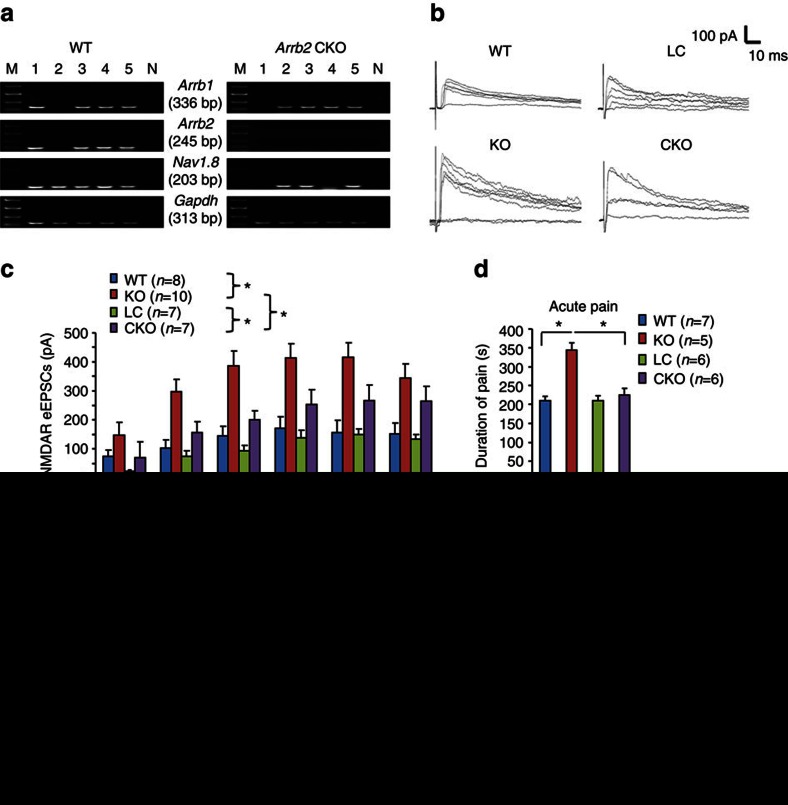
Spinal cord presynaptic *Arrb2* deficiency enhances NMDAR function. (**a**) Single-cell PCR shows the expression of *Arrb1*, *Arrb2*, *Nav1.8* and *Gapdh* mRNA in 5 small-sized DRG neurons of WT and *Arrb2* CKO mice with *Arrb2* deletion in Nav1.8-expressing sensory neurons. Note that *Arrb2* but not *Arrb1* is absent in small-sized DRG neurons of CKO mice. M, molecular weight; N, negative control. *Gapdh* is used as positive control. Gel images have been cropped for presentation. Full size images are presented in Supplementary Fig. 8b. (**b**) Traces of NMDAR-induced eEPSCs in WT, KO, CKO and their littermate control (LC) mice following dorsal root stimulation. (**c**) Amplitude of synaptic currents (dorsal root stimulation-evoked and NMDAR-mediated eEPSCs) of WT and KO mice as well as in CKO mice and LC mice. *P<0.05, Two-Way ANOVA, *n*=7–10 neurons per group. Note that KO mice show larger currents than CKO mice. (**d**) Spontaneous pain (0–10 min) after i.t. NMDA (1 nmol) injection. *P<0.05 versus KO and CKO mice, One-way ANOVA. Note that spontaneous pain is potentiated in KO but not CKO mice. (**e**) Mechanical allodynia after i.t. NMDA (1 nmol) injection. ^$^*P*<0.05, Two-Way ANOVA; *P<0.05, ^#^*P*<0.05 (KO versus CKO), Two-Way ANOVA followed by *post-hoc* Bonferroni test, *n*=5–7 mice per group. Note that allodynia is prolonged in both KO and CKO mice. All data are expressed as mean±s.e.m.

Since Arrb2 is expressed in CGRP-positive presynaptic terminals in SDH (Fig. 5f,g), we further determined a possible role of presynaptic Arrb2 in modulating NMDAR function and pain. To this end, we generated conditional knockout (CKO) mice to delete *Arrb2* selectively in primary sensory neurons expressing the sodium channel subunit Nav1.8, by crossing *Arrb2*-floxed mice with *Nav1.8*-Cre mice^35^. Nav1.8 is expressed primarily in C-fibre nociceptive neurons; it is also present in some myelinated A-fibre neurons^36^. Single-cell PCR analysis in small-sized DRG neurons revealed that majority of WT DRG neurons (4 of 5) express *Arrb2*, and this expression was lost in *Arrb2*-CKO mice (Fig. 7a). For comparison, the expression of *Arrb1* was normal and the expression of *Nav1.8* was partially reduced in CKO mice (Fig. 7a). These single-cell PCR results validated the successful generation of *Arrb2*-CKO mice.

Synaptic NMDA currents in SDH neurons evoked by dorsal root stimulation can be mediated by both presynaptic and postsynaptic mechanisms^37^. We compared dorsal root stimulation-evoked and NMDAR-mediated EPSCs (eEPSCs) in IIo neurons of WT, KO and CKO mice. As compared with KO mice, we found a marked increase in eEPSCs in KO mice (Fig. 7b,c), suggesting that Arrb2 is an inhibitory regulator of NMDAR at spinal nociceptive synapses. Of interest NMDAR-mediated eEPSCs in lamina IIo neurons were also increased in CKO mice, although the magnitude of increase was less than that in *Arrb2* global KO mice (Fig. 7c).

Since presynaptic NMDAR in SDH was implied in pain regulation^38^,^39^, we also compared i.t. NMDA-induced acute and chronic pain in WT, KO, CKO mice. Interestingly, i.t. NMDA-induced acute spontaneous pain was only increased in KO but not CKO mice (Fig. 7d). However, i.t. NMDA-induced mechanical allodynia was prolonged in both CKO and KO mice, despite the KO mice exhibited the longer duration (Fig. 7e). Intraplantar capsaicin induces primary and secondary mechanical allodynia, via respective peripheral and central modulation, respectively^4^. Only the capsaicin-evoked primary mechanical allodynia was potentiated in CKO mice (Supplementary Fig. 6a,b). These results suggest that spinal presynaptic Arrb2 also plays an active role in regulating NMDAR function and pain resolution, although KO mice show more severe defects than CKO mice.

### Spinal cord over-expression of *Arrb2* controls chronic pain

**Figure 8:**
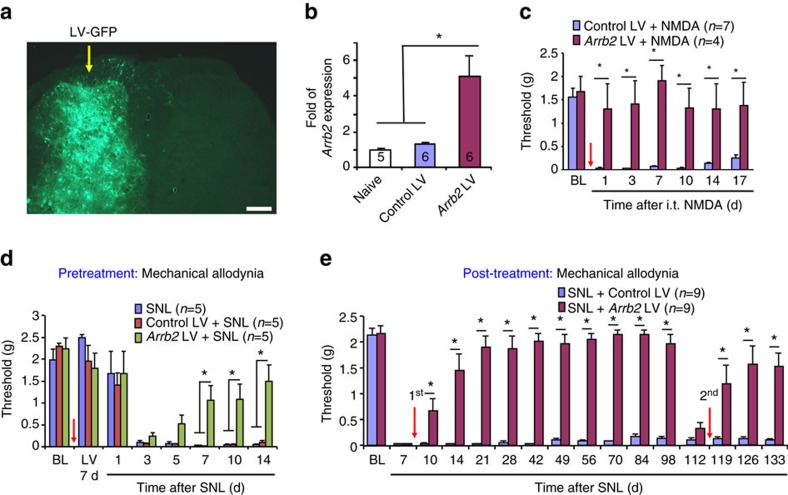
Spinal over-expression of *Arrb2* prevents and reverses neuropathic pain. (**a**) Unilateral SDH microinjections of *Arrb2*-lentivirus (LV) target ipsilateral SDH. Yellow arrow indicates the injection site. Scale, 200 μm. (**b**) *Arrb2* mRNA expression in SDH 2 weeks after the LV injections. *P<0.05, versus naïve control and Control LV, *n*=5–6 mice per group. Following laminectomy, LV injections (2 × 0.4 μl≈10^5^ TU) were made into the L5-SDH via a glass pipette. Scale, 200 μm. (**c**,**d**) Prevention of mechanical allodynia after i.t. NMDA (1 nmol, **c**) and spinal nerve ligation (SNL, **d**) by intra-spinal pretreatment of *Arrb2*-LV, given 7 d before the NMDA injection or SNL. *P<0.05, Two-Way ANOVA. *n*=4–7 mice per group. Arrows indicate the injections. (**e**) Reversal of spinal nerve ligation (SNL)-induced mechanical allodynia by intra-spinal post-treatment of *Arrb2*-LV, given one week after SNL. Note that a second post-treatment (indicated by the red arrow) of LV on day 112 is still effective in reversing mechanical allodynia. *P<0.05, Two-Way ANOVA followed by *post-hoc* Bonferroni test. *n*=9 mice per group. Arrows indicate the injections. All data are expressed as mean±s.e.m.

In addition to loss-of-function approaches in *Arrb2* deficient mice, we also employed a gain-of-function approach to define whether overexpression of *Arrb2* in SDH is sufficient to prevent and resolve chronic pain. Microinjections of *Arrb2*-lentiviral (*Arrb2*-LV) vector into one side of SDH resulted in unilateral virus infection revealed by GFP-labelling, followed by increase in *Arrb2* mRNA levels in SDH (Fig. 8a,b). Double staining of GFP with cellular markers showed that a large number of neurons (NeuN^+^) and some glial cells were infected by LV (Supplementary Fig. 7a) in SDH. We next tested if Arrb2-LV can prevent NMDA and nerve injury-induced persistent pain. Intra-spinal pre-treatment of *Arrb2*-LV, given 14 days before NMDA injection, prevented i.t. NMDA-induced mechanical allodynia for >17 days (Fig. 8c). Spinal nerve ligation (SNL) produced long-lasting neuropathic pain symptoms including heat hyperalgesia (>6 weeks) and mechanical allodynia (>4 months) (Fig. 8d,e; Supplementary Fig. 7b,c). The SNL-induced heat hyperalgesia and mechanical allodynia were effectively prevented by single injection of *Arrb2*-LV (Fig. 8d; Supplementary Fig. 7b). Finally, we assessed whether *Arrb2*-LV can reverse chronic neuropathic pain. Strikingly, posttreatment of *Arrb2*-LV, given 7 days after SNL, completely reversed the SNL-induced heat hyperalgesia (Supplementary Fig. 7c). It also reversed mechanical allodynia for 3 months (Fig. 8e). Furthermore, the second *Arrb2*-LV treatment was still effective in reversing late-phase neuropathic pain (Fig. 8e).

## Discussion

Given a critical role of β-arrestins in regulating GPCR desensitization^11^, *Arrb2* was strongly implicated in inhibiting opioid analgesia^14^,^16^,^17^. Especially, mice lacking *Arrb2* exhibited enhanced and prolonged morphine analgesia^13^. Our result confirmed that i.t. DAMGO-induced acute analgesia is potentiated in *Arrb2*-KO mice. Paradoxically, we also found a prolongation of the late-phase allodynia in KO mice. Recently, it was reported that constitute MOR activity is critical for the resolution of inflammatory pain^9^. However, our results show that enhanced MOR activity in the acute phase is not sufficient to prevent chronic pain development in *Arrb2*-KO mice after DAMGO withdrawal. By contrast, these KO mice exhibit a prolongation of DAMGO-induced allodynia, indicating an active role of Arrb2 in controlling the duration of persistent pain after opioid withdrawal. Apart from GPCR(ref. 11), *Arrb2* has also been shown to regulate non-GPCR targets, such as TRPV1 ion channel^21^ and TGF-β receptors^18^. This is the first study to demonstrate that Arrb2 deficiency leads to increased surface expression and hyperactivity of the NMDAR GluN2B subunit in spinal lamina IIo neurons in association with chronic pain states. Strikingly, *Arrb2*-dificient lamina IIo neurons displayed profound increases in synaptic NMDA currents and also in total NMDA currents that are primarily mediated by GluN2B. Furthermore, we demonstrated possible interaction of Arrb2 with GluN2B but not GluN2A. The ability of NMDA to directly stimulate a pain-like behaviour (licking and allodynia) that is sensitive to Arrb2 suggests that Arrb2 may also act downstream of NMDAR, possibly via a feedback loop. NMDAR activation is known to cause the phosphorylation and activation of extracellular signal-regulated kinase (ERK) in SDH neurons, a critical step for the generation of central sensitization and pain hypersensitivity^6^. Future study is needed to investigate how NMDAR, ERK and Arrb2 interact with each other.

Our data also showed that Arrb2 regulation of NMDAR function is cell-specific: it was only observed in spinal lamina IIo neurons but not in spinal lamina I neurons and hippocampal CA1 neurons. As an important output of spinal cord nociceptive processing, lamina I projection neurons have been extensively investigated in pain research^31^,^40^. However, the importance of lamina IIo excitatory interneurons has begun to be revealed^25^,^27^. Recent evidence suggests these interneurons are critically required for mediating mechanical pain, a cardinal feature of chronic pain^25^. Lamina IIo interneurons form a nociceptive circuit by receiving direct input from C-fibres and sending output to lamina I projection neurons^24^,^41^, integrate various nociceptive input in SDH^27^, and exhibit marked plastic changes in chronic pain conditions^42^,^43^. Since there is a universal expression of Arrb2 in SDH, lack of NMDAR modulation by Arrb2 in lamina I neurons should not be a result of loss of Arrb2 in these neurons. It is more likely there is lack of functional coupling between Arrb2 and NMDAR in lamina I neurons. In support of a region-specific modulation of NMDAR function by Arrb2, *Arrb2*-deficient mice exhibit normal LTP but impaired NMDA-dependent long-term depression in the hippocampus, associated with spatial learning deficits^44^. It was also suggested that MOR activity is differentially regulated in diverse regions of the central nervous system (spinal cord versus brain) in *Arrb2*-KO mice^16^. It remains to be tested whether there is direct interaction between Arrb2 and NMDAR in lamina IIo neurons. Arrb2 may indirectly interact with GluN2B via other proteins.

Our data also showed that Arrb2 regulates spinal cord NMDAR/GluN2B function not only at postsynaptic sites but also at extrasynaptic and presynaptic sites, since both synaptic and total NMDA currents were increased in lamina IIo neurons after loss of Arrb2. GluN2B subunits are known to be localized at extrasynaptic sites and play an important role in neurodegeneration and synaptic plasticity in pathological conditions^45^. We also demonstrated a presynaptic regulation of NMDAR function by Arrb2, as synaptic NMDA currents in lamina IIo neurons were enhanced in CKO mice with Arrb2 deficiency in Nav1.8-expressing presynaptic terminals. Furthermore, i.t. NMDA-induced and GluN2B-mediated mechanical allodynia was also prolonged in CKO mice. Consistently, it has been shown that GluN2B-mediated presynaptic NMDAR activity in the primary sensory afferents plays an active role in enhanced glutamatergic response in the spinal first sensory synapse after peripheral nerve injury^46^. Additionally, Arrb2 can also control the duration of inflammatory pain via peripheral mechanisms such as desensitization of TRPV1 in DRG neurons^21^. Indeed, intraplantar capsaicin-induced primary allodynia was potentiated in *Arrb2*-CKO mice (Supplementary Fig. 6).

Another important but surprising finding of this study is that Arrb2 is indispensable for the resolution of all the inflammatory and neuropathic pains we tested in this study, despite the fact that Arrb2 is a multifunctional scaffold protein that can target numerous surface molecules (receptors and channels) exerting both pronociceptive (for example, TRPV1)^21^ and antinociceptive (for example, opioid receptors)^13^ actions. It is important to point out that the overall net effect of Arrb2 is to suppress pain or ‘arrest pain’ after inflammation and nerve injury. Thus, loss of Arrb2 resulted in a prolongation of inflammatory and neuropathic pains, without affecting the baseline pain. Strikingly, a single i.t. application of NMDA was sufficient to induce chronic mechanical allodynia in *Arrb2*-KO mice but not WT mice. Conversely, spinal over-expression of *Arrb2* via lentivirus effectively reversed established neuropathic pain, even the lentivirus was given 3 months after nerve injury.

A central question in pain research is what causes the transition from acute to chronic pain. Over the last several decades, our knowledge about how pain is induced by various inflammatory mediators has been greatly expanded and deepened^1^. However, our understanding of mechanisms underlying pain transition and resolution is still very limited^9^,^47^,^48^,^49^. A failure in the resolution of acute pain will lead to the transition from acute pain to chronic pain^8^. Levine and collaborates proposed hyperalgesic priming, a form of neuroplasticity in nociceptors, as a model of the transition from acute to chronic pain^50^. In this model, subsequent injection of prostaglandin E2 (PGE2), after the resolution of acute pain by an initial insult, produces marked prolongation of mechanical hyperalgesia, which involves IB4-binding nociceptors, protein kinase ε (PKCε) and protein translation^50^,^51^. Of interest inflammation downregulates the GRK2 expression in DRG neurons and knockdown of the GRK2 expression in naïve animals led to a prolonged hyperalgesia induced by multiple inflammatory mediators including PGE2 (refs. 10, 48, 52). GRK2 mediates the transition from acute to chronic inflammatory pain via biased cAMP signalling to EPAC1 (exchange protein directly activated by cAMP), PKCε and ERK/MAP kinase^52^. Further studies are required to invesitgate the assoication of Arrb2 with GRK2, PKCε, EPAC and ERK in primary sensory neruons.

In SDH several mechanisms have been proposed for the maintenance of chronic pain. Protein kinase M-zeta (PKMζ) was involved in the maintenance of persistent nociceptive sensitization^53^. Tissue inflammation also produces latent pain sensitization that is masked by spinal MOR signalling for months, and blocking endogenous MOR causes chronic pain via NMDAR-mediated activation of calcium-sensitive adenylyl cyclase type 1 (refs. 9, 54). Hyperalgesia and spinal LTP can be rendered labile at the spinal level and erased following reactivation in a process analogous to memory reconsolidation^32^,^49^. Spinal LTP and persistent pain may also be erased by high dose of opioid^55^. It will be of great interest to examine how Arrb2 is associated with these spinal cord mechanisms for the maintenance and resolution of chronic pain. Importantly, spinal over-expression of *Arrb2* is sufficient to reverse chronic neuropathic pain.

In summary, using both loss-of-function (Arrb2-KO mice) and gain-of-function (Αrrb2 over-expression) strategies, we demonstrate that Αrrb2 in SDH contributes to the transition of acute pain to chronic pain. Loss of Arrb2 leads to a marked prolongation of inflammatory and neuropathic pain, as well as i.t. NMDA-induced allodynia. Mechanistically, Arrb2 controls the transition from acute to chronic pain via suppressing the activity of NMDAR/GluN2B in spinal lamina IIo neurons. Emerging evidence suggests that disinhibition—loss of GABAergic and glycinergic transmission in spinal pain circuit—is a powerful mechanism for the transition from acute to chronic pain^56^,^57^,^58^. Chronic pain syndromes may also result from a loss of endogenous analgesic control^54^. We discovered that neuronal and synaptic plasticity in spinal cord lamina IIo can also be regulated by Arrb2 via a mechanism that is GRCR-independent but NMDAR-dependent. Thus, Arrb2 may serve as an intracellular gate keeper in spinal cord pain circuit and contributes to the resolution of chronic pain. Targeting spinal Arrb2 signalling may shed light on the development of new therapeutics for the prevention and treatment of chronic pain.

## Methods

### Reagents

We purchased capsaicin, carrageenan, complete Freund’s adjuvant (CFA), paclitaxel, formalin, NMDA, GABA, GDPβS (G-protein inhibitor), DAMGO, MK-801 from Sigma-Aldrich, TCN201 (GluN2A antagonist), Ro25-6981 (GluN2B antagonist), DHPG (group I metabotropic glutamate receptor agonist) from Tocris.

### Animals

*Αrrb2* global KO mice and *Αrrb2*^flox^ mice (both with C57BL/6 background) were from laboratories of Robert Lefkowitz and Wei Chen at Duke University Medical Center and maintained at Duke animal facility. All mice were housed (2–5 mice per cage) in a standard 12:12 light–dark cycle with normal illumination. To selectively delete *Αrrb2* in Nav1.8-expressing nociceptive/primary sensory neurons^36^, we crossed mice carrying a conditional null allele of *Αrrb2* (*Αrrb2*^f/f^) with *Nav1.8*^cre^ transgenic mice (kindly provided by Rohini Kuner, University of Heidelberg), and the resulting homozygous CKO mice (*Αrrb2*^f/f^; *Nav1.8*^cre^) are referred to CKO mice. The *Αrrb2*^f/f^ littermates were used as littermate control (LC) mice. Breeding colonies were maintained by mating *Αrrb2*^f/f^ with *Nav1.8*^*cre*^-*Αrrb2*^f/f^ mice. *Nav1.8*^cre^ mice had been backcrossed for at least 7 generations on a C57BL/6 background. Mice were genotyped by PCR using genomic DNA isolated from ear according to standard protocols from the Jackson Labs. Adult male mice (8–10 weeks) were used for behavioural and biochemical studies. Young mice (4–7 weeks) were used for electrophysiological studies in spinal cord slices to obtain high quality recordings. For lentivirus injection experiment, adult ICR (male, 25–35 g) were purchased from the Experimental Animal Center of Nantong University. All animals were housed under a 12-hour light/dark cycle with food and water available *ad libitum*. No statistical method was used to predetermine sample size. No randomization was applied to the animal experiments. Sample sizes were estimated based on our previous studies for similar types of behavioural, biochemical and electrophysiological analyses^26^,^34^,^42^. All the animal procedures were conducted in accordance with the National Institutes of Health Guide for the Care and Use of Laboratory Animals and approved by the Institutional Animal Care & Use Committee (IACUC) of Duke University and Nantong University. The number of animals used in each experiment was described in Supplementary Table 1.

### Animal models of pain and i.t. injection

To produce inflammatory pain, diluted formalin (5%, 20 μl) or carrageenan (1.5%, 20 μl) was injected into the plantar surface of a hindpaw. Intraperitoneal injection of paclitaxel (6 mg kg^−1^, i.p.) was given to generate chemotherapy-associated neuropathic pain^43^,^59^. Neuropathic pain was also induced by SNL (ref. 60). CD1 mice were anaesthetised with isoflurane and the L6 transverse process was removed to expose the L4 and L5 spinal nerves. The L5 spinal nerve was then isolated and tightly ligated with 6-0 silk thread. For sham operations, the L5 spinal nerve was exposed without ligation. For i.t. injection, spinal cord puncture was made with a 30G needle between the L5 and L6 level to deliver reagents (10 μl) to the cerebral spinal fluid^61^. The doses of DAMGO, NMDA, MK801, TCN201, Ro25-6981 for behavioural and electrophysiological experiments were chosen on the basis of previous reports^6^,^9^,^38^,^62^,^63^. The doses were also chosen based on manufacturer’s instructions and our preliminary studies.

### Spinal injection of lentiviral vector

To determine a critical role of Arrb2 in pain resolution, we employed a gain-of-function strategy via gene therapy using lentiviral (LV) vector. Replication-deficient LV vector carrying the gene of *Arrb2* (*Arrb2*-LV) and control LV vector were generated by GeneChem (Shanghai, China), and the final tilter of *Arrb2*-LV is 1 × 10^8^ TU ml^−1^. These vectors also contain GFP for tracing virus infection. Intra-spinal injections were performed as we recently demonstrated^25^. Hemi-laminectomy was performed on the ipsilateral spinal cord at L4-L5 vertebral segments, and each animal was given two ipsilateral injections (2 × 0.4 μl, 0.8 mm apart and 0.5 mm deep) of *Arrb2*-LV or control-LV (1 × 10^5^ TU) using a glass micropipette (diameter of 80 μm). These injections were made 7 days before SNL, 7 days after SNL, or 112 days after SNL surgery. For some mice, multiple injections (6 × 0.4 μl, 0.8 mm apart and 0.5 mm deep) were made at L3-L6 SDH 7 days before the i.t. NMDA injection. The tip of glass micropipette reached to the depth of lamina II–IV of the SDH.

### Behavioural testing

Animals were habituated to the environment for at least 2 days before the testing. All the behaviours were tested blindly. We assessed formalin-evoked spontaneous inflammatory pain by measuring the time (seconds) mice spent on licking and lifting the affected paw every 5 min for 90 min (ref. 64). For testing mechanical sensitivity, we confined mice in boxes placed on an elevated metal mesh floor and stimulated the hindpaws with a series of von Frey hairs with logarithmically increasing stiffness (0.02–2.56 g, Stoelting), presented perpendicularly to the central plantar surface. We determined the 50% paw withdrawal threshold by Dixon’s up-down method. Thermal sensitivity was tested using hot plate and Hargreaves radiant heat apparatus (IITC Life Science). For the radiant heat test, the basal paw withdrawal latency was adjusted to 9–12 s, with a cutoff of 20 s to prevent tissue damage. For testing cold allodynia, a drop of acetone was applied to the ventral surface of a hind paw and the mouse’s response was observed for 30 s after acetone application. Responses to acetone were graded according to the following 4-point scale: 0, no response; 1, quick withdrawal or flick of the paw; 2, prolonged withdrawal or flicking; 3, repeated flicking with licking^65^.

### Spinal cord slice preparation and patch clamp recordings

As we previously reported^26^,^34^, a portion of the lumbar spinal cord (L4-L5) was removed from mice (4–7 weeks old) under urethane anaesthesia (1.5–2.0 g/kg, i.p.) and kept in pre-oxygenated ice-cold Krebs solution. Transverse slices (400–600 μm) were cut on a vibrating microslicer. The slices were perfused with Kreb’s solution (8–10 ml min^−1^) that was saturated with 95% O_2_ and 5% CO_2_ at 26 °C for at least 1–3 h prior to experiment. The Kreb’s solution contains (in mM): NaCl 117, KCl 3.6, CaCl_2_ 2.5, MgCl_2_ 1.2, NaH_2_PO_4_ 1.2, NaHCO_3_ 25 and glucose 11.

The whole cell patch-clamp recordings were made from lamina IIo neurons in voltage clamp mode as we previously reported^34^. Patch pipettes were fabricated from thin-walled, borosilicate, glass-capillary tubing (1.5 mm o.d., World Precision Instruments). The resistance of a typical patch pipette is 5–10 MΩ. Membrane currents were amplified with an Axopatch 700B amplifier (Axon Instruments) in voltage-clamp mode. Signals were filtered at 2 kHz and digitized at 5 kHz. Data were stored with a personal computer using pCLAMP 10 software and analysed with Mini Analysis (Synaptosoft Inc.).

To measure evoked EPSCs (eEPSCs) in lamina IIo neurons, dorsal root enter zone was stimulated through a concentric bipolar electrode (FHC) with an isolated current stimulator^34^. The internal solution contains (in mM): Cs_2_SO_4_ 110, CaCl_2_ 0.1, MgCl_2_ 2, EGTA 1.1, HEPES 10 and ATP-Mg 5. After establishing the whole-cell configuration, neurons were held at the potential of −70 mV to record eEPSCs. QX-314 (5 mM) was added to the pipette solution to prevent discharge of action potentials. Test pulses of 0.1 ms with intensity of 0.5–3 mA were given at 30 s intervals and monosynaptic C-fibre responses were recorded. The responses were considered as monosynaptic in origin when the latency remained constant and there was no failure during stimulation at 20 Hz for 1 s, or when failures did not occur during repetitive stimulation at 2 Hz for 10 s (refs. 66, 67). Synaptic strength was quantified by the peak amplitudes of eEPSCs. To measure NMDAR-mediated eEPSC, recording electrodes had resistances of 4–8 mV after being filled with an internal solution. The spinal cord slice was kept in the holding chamber for at least 1 h before being transferred to the recording chamber. The slice was transferred into a holding chamber containing normal Mg^2+^-free ACSF with 2 μM CNQX bubbled with 95% O_2_ and 5% CO_2_ at 26 °C. After establishing the whole-cell configuration, neurons were held at the potential of +40 mV to record NMDAR-mediated eEPSCs. Total NMDA currents were also recorded in IIo neurons by perfusing spinal cord slices with 50 μM NMDA for 30 s. As positive controls for G protein inhibition, currents were also induced by bath application of DHPG (50 μM) and GABA (1 μM), when neurons were held at −70 and 0 mV, respectively. The internal solution for recording DHPG-induced currents contains (in mM): potassium gluconate 135, KCl 5, CaCl_2_ 0.5, MgCl_2_ 2, EGTA 5, HEPES 5 and ATP-Mg 5. The internal solution for GABA-induced currents contains (in mM): Cs_2_SO_4_ 110, CaCl_2_ 0.1, MgCl_2_ 2, EGTA 1.1, HEPES 10 and ATP-Mg 5. Apart from spinal lamina IIo neurons, total NMDA currents were also recorded in lamina I neurons of spinal cord slices and hippocampal CA1 neurons of brain slices after bath application of NMDA (50 μM, 30 s). Spinal cord lamina I projection neurons were validated by their responses to substance P (1 μM). Brain slices (400μm) were prepared in a way similar to spinal cord slices.

### *In situ* hybridization

Digoxigenin (DIG)-labelled RNA probes were used for *in situ* hybridization. For generating the *Arrb2* antisense probe, mouse cDNA fragment was amplified by PCR with the antisense primer containing the T7 promoter sequence. The sequences of the primers for antisense probe were as follows:

*Arrb2*-F: AGAAAAACCCGGGACCAG, *Arrb2*-R: GATCCCCAGCACCTCCTT.

*In vitro* transcription was then performed from the PCR-amplified template using T7 or sp6 RNA polymerase (Roche) with Digoxigenin-UTP (Roche) for the synthesis of the antisense probe. Spinal cord sections (20 μm) were used for *in situ* hybridization^68^. Pre-hybridization, hybridization and washing were performed according to standard methods. Spinal cord sections were then incubated with alkaline phosphatase-conjugated anti-Digoxigenin antibody (1:3,500; Roche) for overnight at 4 °C. After washing, the *in situ* signals were developed with Fast Red substrate (Roche). For further immunohistochemistry, spinal cord sections were blocked with 1% BSA for 1 h at room temperature. The sections were then incubated overnight at 4 °C with the following primary antibodies: NeuN antibody (1:1,000, mouse, Millipore, catalogue #MAB377), GFP antibody (1:500, rabbit, Abcam, catalogue #ab6556). The sections were then incubated for 1 h at room temperature with FITC-conjugated secondary antibodies (1:400; Jackson ImmunoResearch).

### Immunohistochemistry

After appropriate survival times, animals were deeply anaesthetised with isoflurane and perfused through the ascending aorta with PBS, followed by 4% paraformaldehyde with 1.5% picric acid in 0.16 M phosphate buffer. After the perfusion, the L4/L5 DRGs and L4–L5 spinal cord segments were removed and postfixed in the same fixative overnight. DRG sections (14 μm) and spinal cord sections (30 μm, free-floating) were cut in a cryostat. The sections were first blocked with 2% goat or horse serum for 1 h at room temperature. The sections were then incubated overnight at 4 °C with the following primary antibodies: Arrb2 antibody (1:200, rabbit, Cell signalling, catalogue #3857), CGRP antibody (1:1,000, goat, Abcam, catalogue #ab36001), NeuN antibody (1:1,000, mouse, Millipore, catalogue #MAB377), GFP Antibody (1:500, rabbit, Abcam, catalogue #ab6556), GFAP antibody (1:1,000, mouse, Millipore, catalogue #MAB360), and IBA-1 antibody (1:1,000, rabbit, Wako Chemicals, catalogue #019-19741). The sections were then incubated for 1 h at room temperature with cyanine 3 (Cy3)- or FITC-conjugated secondary antibodies (1:400; Jackson ImmunoResearch). For double immunofluorescence, sections were incubated with a mixture of polyclonal and monoclonal primary antibodies, followed by a mixture of Cy3- and FITC-conjugated secondary antibodies. The stained and mounted sections were examined with a Nikon fluorescence microscope, and images were captured with a CCD Spot camera. Some sections were also examined under a Zeiss 510 inverted confocal microscope.

### Cell culture and transfection

We chose Hela cells because these cells have low basal expression of βarr2, compared with HEK293 cells. The Hela cell line was obtained from cell culture facility of Duke University. Cells were cultured in high glucose (4.5 g l^−1^) Dulbecco’s Modified Eagle’s Medium containing 10% (v/v) fetal bovine serum. Transfection (2 μg cDNA) was performed with LipofectamineTM 2000 Reagent (Invitrogen) at 70% confluency and the transfected cells were cultured in the same medium for 48 h before biochemical studies. NR1-YFP, NR2A-SEP, NR2B-SEP and Arrb2-flag pcDNA3, as well as Arrb2-GFP plasmid were obtained from Addgene.

### Immunoprecipitation

HeLa cells and SDH tissues were lysed in RIPA buffer (Milipore) and mechanically homogenized. The lysates were incubated on ice overnight with occasional shaking with 0.1 μg anti-flag antibody (mouse, Sigma), 1 μg anti-GFP antibody (rabbit, Invitrogen) for HeLa cell proteins, and 1 μg anti-NR2A/GluN2A antibody (mouse, NeuroMab, catalogue #75-288), 1 μg anti-NR2B/GluN2B antibody (mouse; NeuroMab, catalogue #75-097), and 1 μg anti-Arrb2 antibody (rabbit; cell signalling, catalogue #3857) for spinal cord proteins. The antibody-protein complexes were aggregated by Protein G-Agarose (Pierce) on ice overnight, with occasional shaking, and then centrifuged at 8000*g* for 10 min. The pellet was washed for elimination of non-specific binding with × 1 RIPA buffer and eluted by × 4 SDS sample buffer without DTT and boiled for 10 min and then processed for western blot.

### Biotinylation for surface proteins

Plasma membrane protein expression was detected after protein biotinylation. Briefly, 0.5 mg ml^−1^ EZ-Link sulfo-NHS-LC-biotin (Pierce) was added to transfected cells, and the mix was incubated on ice for 30 min, with occasional shaking. The biotinylation reaction was terminated and washed in Ca^2+/^Mg^2+^ PBS solution and cells were harvested. Cells were washed out and solubilized in PBS containing 1% triton X-100, 5 mM EGTA, 5 mM EDTA, 50 mM NaF, 10 mM Na-Pyrophophate, 1 mM NaVO_3_, and protease inhibitor cocktail (Sigma). Solubilized lysates were pulled down on Streptavidin Agarose Resin (Pierce) overnight on ice, with occasional shaking. The pellet was eluted by × 4 sample buffer at boiling temperature. All lysate proteins were quantitated by BCA assay for western blot.

### Preparation of synaptosome-containing P2 fractions

As previously described^29^, SDH tissue (∼50–100 mg) was homogenized in 10 volumes of the Syn-PER Reagent (Thermo Scientific) using a 1 mL pipette for 20 up-and-down strokes. The homogenate was centrifuged at 1,200*g* for 10 min to remove cell debris, and the supernatant was centrifuged at 15,000*g* for 20 min. The synaptosomes containing pellets were gently suspended in the RIPA lysis buffer (× 10, Millipore) containing protease and phosphatase inhibitors for further Western blot analysis.

### Western blot

Proteins from tissues and cell cultures (20–50 μg) were separated on SDS-PAGE gel (4–15%; Bio-Rad)^34^. After the transfer, the blots were incubated overnight at 4 °C with polyclonal antibody against Arrb2 (1:1,000, rabbit, cell signalling, catalogue #3857), PSD-95 (1:1,000, mouse, Millipore, catalogue #1598), synaptophysin (1:500, mouse; Millipore, catalogue #MAB368), NR2A/GluN2A (1:1,000, mouse, NeuroMab, catalogue #75-288), NR2B/GluN2B (1:1,000, mouse, NeuroMab, catalogue #75-097), N-cadherin (1:1,000, mouse, sigma, catalogue #c3865), Flag (1:5,000, mouse, sigma, catalogue #f3165), and GFP (1:1,000, Abcam, catalogue #ab6556). For loading control, the blots were probed with beta tubulin (β-TUB) antibody (1:5,000, mouse; Millipore, catalogue #MAB3408). These blots were further incubated with HRP-conjugated secondary antibody, developed in ECL solution (Pierce), and the chemiluminescence was revealed by Bio-Rad ChemiDoc XRS for 1–5 min. Specific bands were evaluated by apparent molecular sizes. The intensity of the selected bands was analysed using NIH Image J software. Western gel images have been cropped for presentation. Full size gel images are presented in Supplementary Figs 8a-c.

### Primary cultures of DRG neurons and single-cell PCR

We aseptically removed DRGs from 4 to 5 week-old mice and digested the tissues with collagenase (1.25 mg ml^−1^, Roche) and dispase-II (2.4 units ml^−1^, Roche) for 90 min, followed by 0.25% trypsin for 8 min at 37 °C. We plated cells on slide chambers coated with poly-D-lysine and laminin or on plates coated with poly-D-lysine and grew them in a neurobasal defined medium (with 2% B27 supplement) in the presence of 5 μM AraC, at 37 °C, with 5% CO_2_/95% air for 24 h before experiments. A single cell was aspirated into a patch pipette, gently put into a reaction tube containing reverse transcription reagents, and incubated for 1 h at 50 °C (superscript III, Invitrogen)^66^. The cDNA product was then used in separate PCR. The sequences of all the primers used for single-cell PCR are described in Supplementary Table 2. The 1st and 2nd round PCR was performed using ‘outer’ primers and ‘inner’ primers, respectively. A negative control was obtained from pipettes that did not harvest any cell contents. The PCR products were displayed on ethidium bromide-stained 1.5% agarose gels. Gel images have been cropped for presentation, and full size images are presented in Supplementary Fig. 8b.

### Statistical analyses

All data were expressed as mean±s.e.m. Biochemical, behavioural and electrophysiological data were analysed using Student’s *t*-test (two groups) or Two-Way or One-Way ANOVA followed by *post-hoc* Bonferroni test^26^,^43^. The criterion for statistical significance was *P*<0.05.

### Data availability

The data that support the findings of this study are available from the corresponding author on request.

## Additional information

**How to cite this article:** Chen, G. *et al.* β-arrestin-2 regulates NMDA receptor function in spinal lamina II neurons and duration of persistent pain. *Nat. Commun.* 7:12531 doi: 10.1038/ncomms12531 (2016).

## Supplementary Material

Supplementary Information Supplementary Figures 1-8 and Supplementary Tables 1 & 2.
