# Supplementary material for: β-arrestin-2 regulates NMDA receptor function in spinal lamina II neurons and duration of persistent pain
Source: Nat Commun. 2016 Aug 19;7:12531. doi: 10.1038/ncomms12531 (PMC5477285; doi:10.1038/ncomms12531)
Supplement: Supplementary Information — Supplementary Figures 1-8 and Supplementary Tables 1 & 2. [file ncomms12531-s1.pdf]

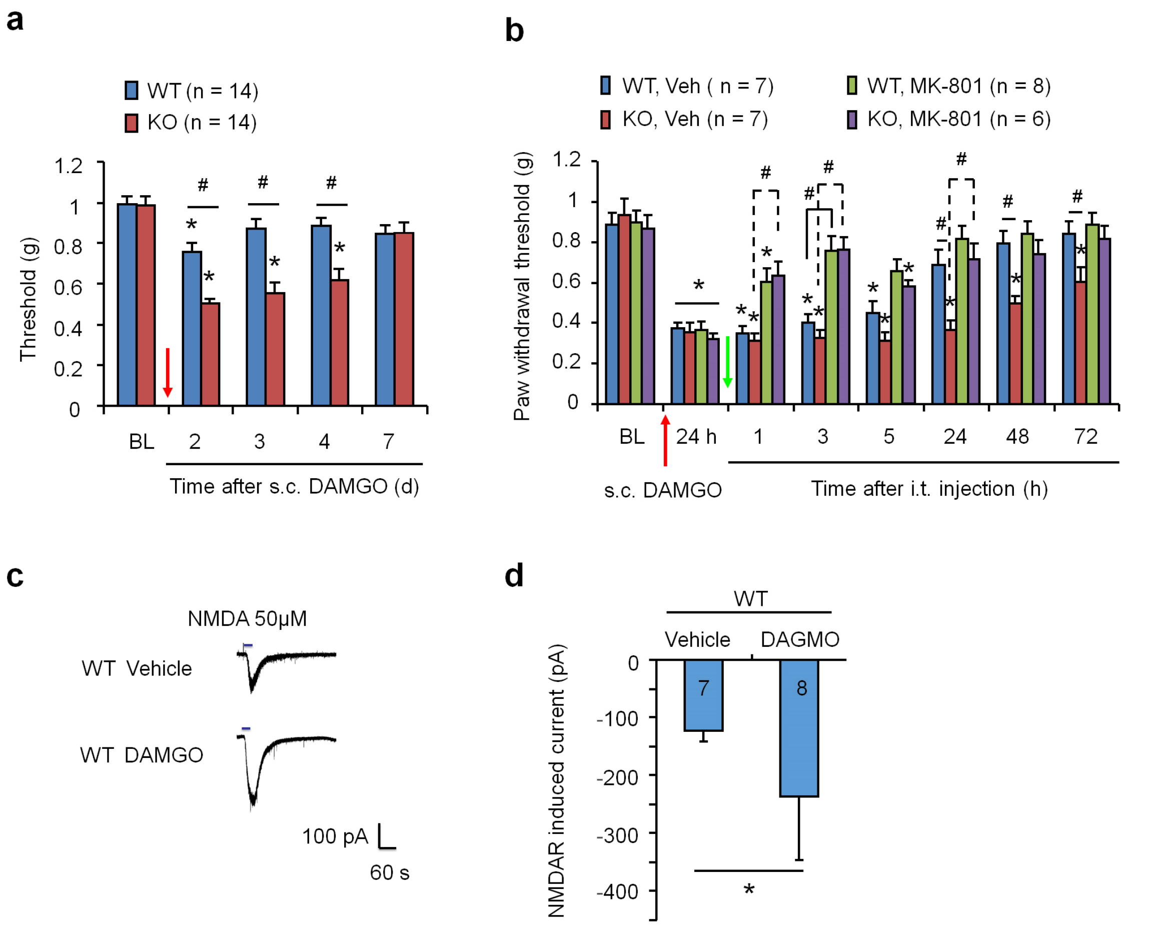

Supplementary Figure 1. Mechanical allodynia and spinal NMDA currents in WT and *Arrb2* KO mice

(**a**) Mechanical allodynia following DAMGO injection (1 mg/kg, s.c.) is potentiated and prolonged in *Arrb2*-KO mice. **P*<0.05, vs. baseline (BL), $^{\#}P$<0.05, vs. WT, Two-Way ANOVA. n = 14 mice/group. (**b**) Subcutaneous DAMGO (1 mg/kg, i.p.) induced mechanical allodynia is blocked by spina administration of NMDAR antagonist MK-801 (i.t., 10 nmol) in WT and KO mice. **P*<0.05, vs. BL, $^{\#}P$<0.05. Two-Way ANOVA, n = 6-8 mice/group. (**c, d**) NMDA-induced current in lamina IIo neurons of spinal cord slices is enhanced in WT mice 24 h after DAMGO treatment. (**c**) Representative traces of NMDA (50 μM) induced current in lamina IIo neurons from vehicle (saline) treated WT mice at 24 h. (**d**) Amplitude of NMDA-induced currents in WT mice. **P*<0.05, student's t-test, n = 7, 8 neurons/group (shown in each column). All data are expressed as mean ± S.E.M.

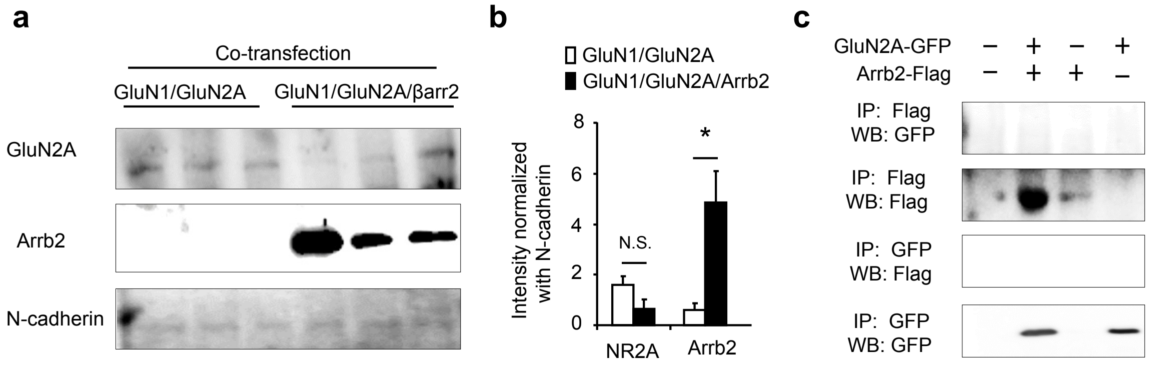

Supplementary Figure 2. Arrb2 is not associated with GluN2A in Hela cells

(**a**) Surface expression of GluN2A and Arrb2 in HeLa cells transfected with GluN1/GluN2A and GluN1/GluN2A/Arrb2. Note that Arrb2 does not regulate GluN2A surface expression in Hela cells. (**b**) Relative expression levels of GluN2A and Arrb2, normalized with N-cadherin. *$P<0.05$, student's t-test, N.S., no significance. n = 3 cultures/group. (**c**) Absence of co-IP of Arrb2 with GluN2A in Hela cells. All data are expressed as mean ± S.E.M.

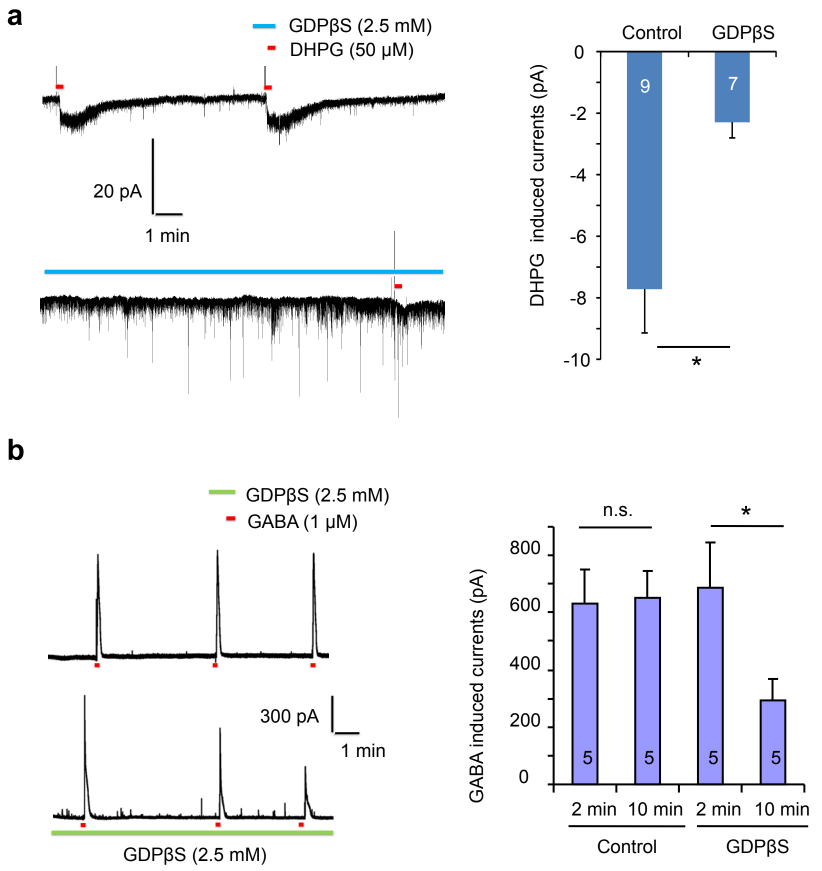

Supplementary Figure 3. Effects of GDPβS on DHPG and GABA-induced currents in spinal lamina IIo neurons of WT mice.

(**a**) Inhibition of mGluR agonist DHPG (50 μM) induced inward currents by GDPβS (2.5 mM) through intracellular delivery via a recording pipette. Right, amplitude of DHPG-induced currents before and after GDPβS treatment. **P*<0.05, student's t-test, n = 7, 9 neurons/group. (**b**) Inhibition of GABA (1 μM) induced outward currents by GDPβS (2.5 mM) through intracellular delivery via a recording pipette. Right, amplitude of GABA-induced currents. **P*<0.05, student's t-test. N.S., no significance. n = 5 neurons/group. Note there is time-dependent inhibition of GABA current. GDPβS effectively inhibits G-protein signaling in lamina IIo neurons of spinal cord slices following bath application of DHPG and GABA. All data are expressed as mean ± S.E.M.

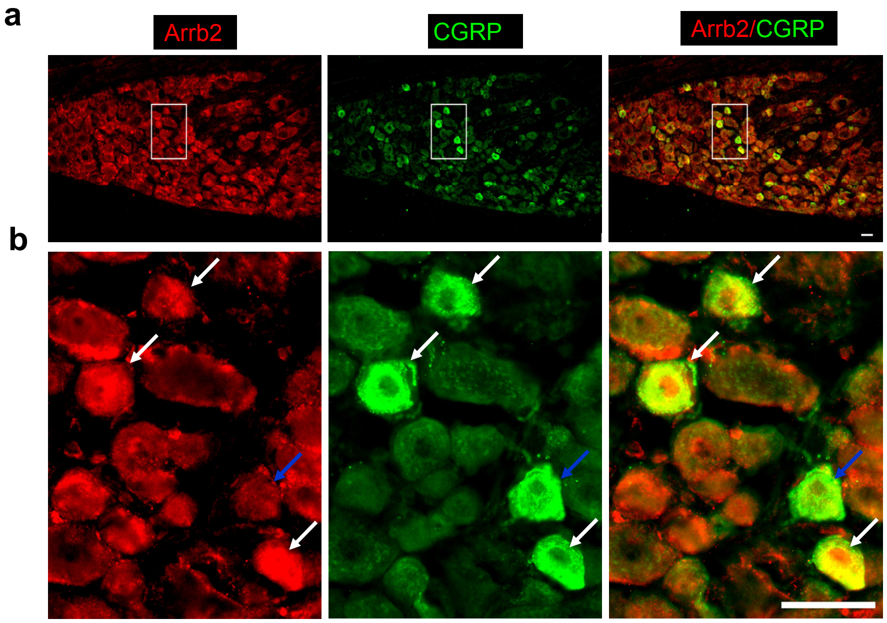

Supplementary Figure 4. Arrb2 expression in DRG sensory neurons of rats.

(**a**) Double staining shows colocalization of Arrb2 and CGRP in DRG neurons. Scales, 20 µm. (**b**) Enlarged boxes from a. White arrows show double-labeled neurons. Scale, 50 µm.

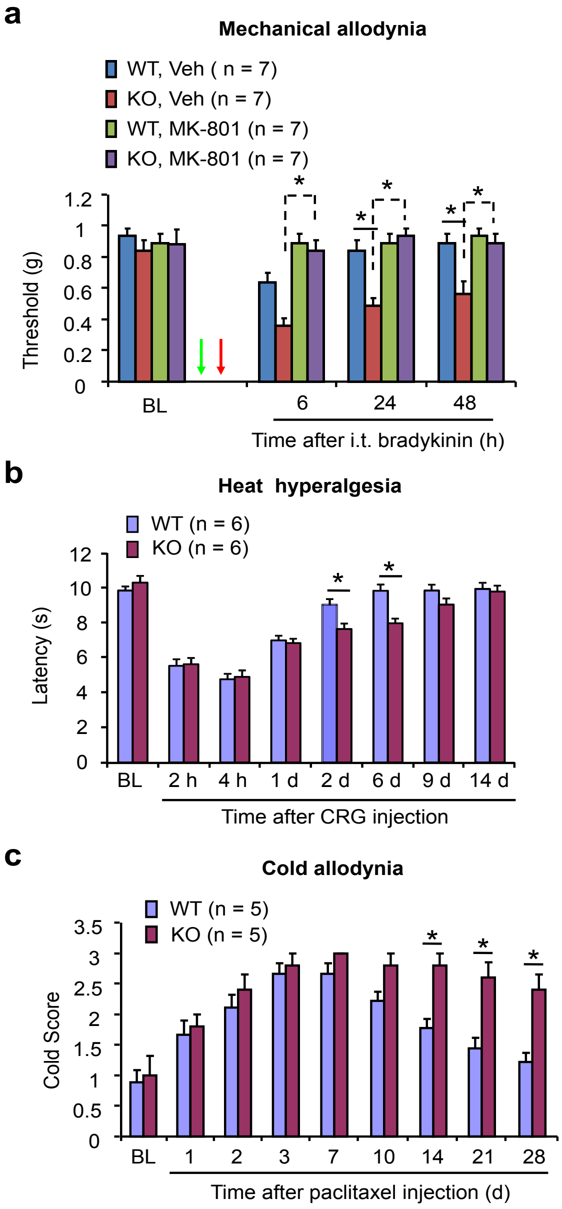

Supplementary Figure 5. Inflammatory and neuropathic pain in WT and *Arrb2* KO mice.

(a) Mechanical allodynia by i.t. bradykinin (1 μg, indicated by red arrow) is enhanced and prolonged in KO mice and prevented by blocking NMDAR with MK-801 (i.t., 10 nmol). $*P<0.05$, two-way ANOVA, n = 7 mice/group. (**b**) Carrageenan-induced heat hyperalgesia is prolonged in KO mice. $*P<0.05$, two-way ANOVA, n = 6 mice/group. (**c**) Paclitaxel-induced cold allodynia is also prolonged in KO mice $*P<0.05$, Two-Way ANOVA, followed by post-hoc Bonferroni test, n = 5 mice/group. All data are expressed as mean ± S.E.M.

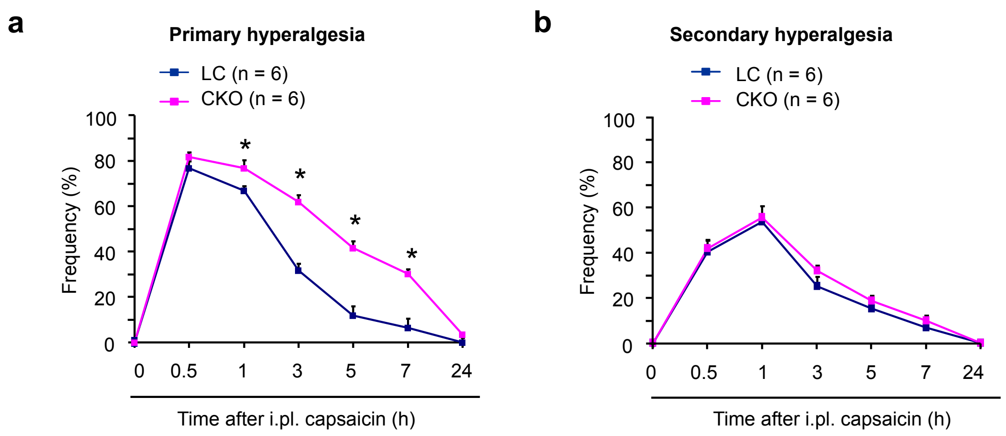

Supplementary Figure 6. Capsaicin-induced 1st and 2nd hyperalgesia/allodynia in WT mice and *Arrb2* conditional knockout (CKO) mice.

(**a, b**) Intraplantar capsaicin-evoked primary mechanical allodynia (a) but not secondary mechanical allodynia (b) is enhanced in CKO mice. LC, littermate control. *P<0.05, vs. LT mice, two-way ANOVA, followed by post-hoc Bonferroni test. n = 6 mice/group. All data are expressed as mean ± S.E.M.

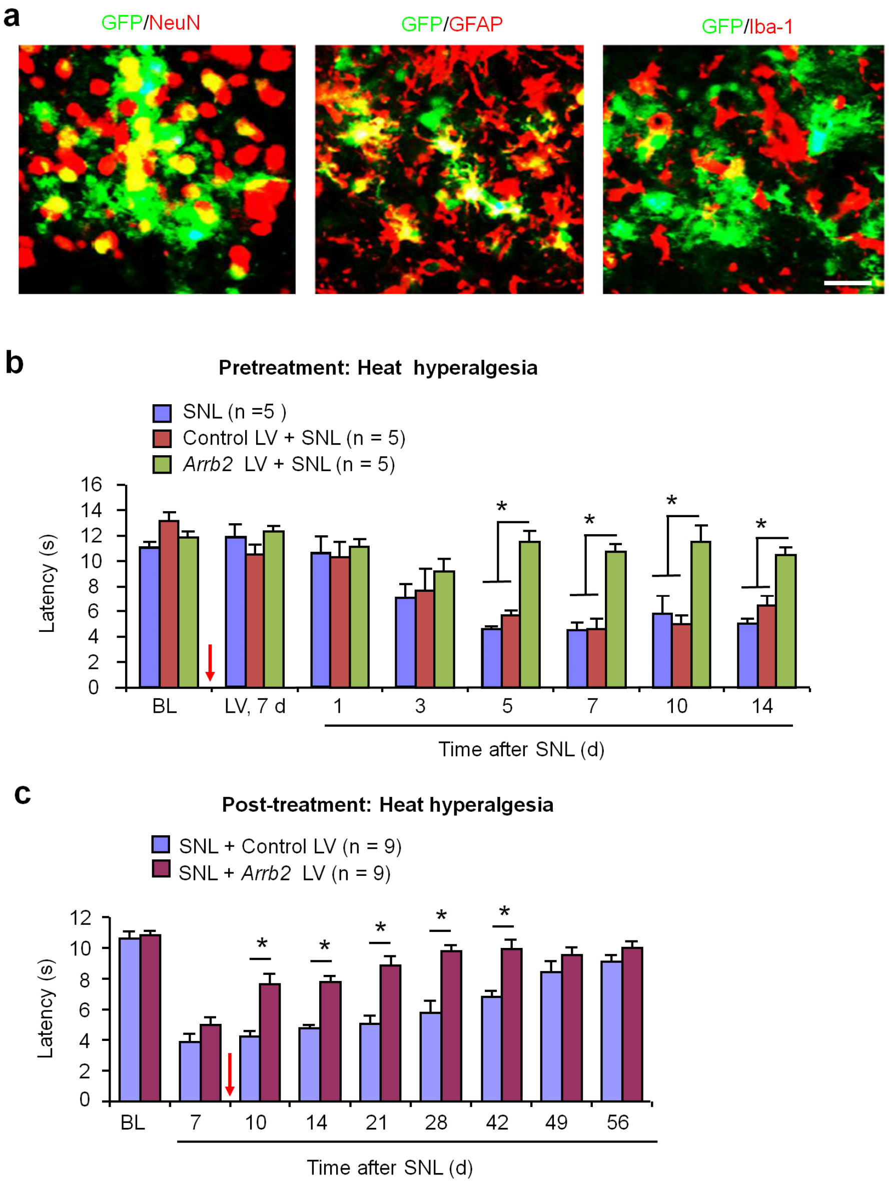

Supplementary Figure 7. Overexpression of *Arrb2* in SDH prevents and reverses heat hyperalgesia in WT mice after SNL

(**a**) Spinal microinjections of GFP-labeled lenti virus (LV) infect many neurons ($NeuN^+$), some astrocytes ($GFAP^+$), and few microglia ($Iba\text{-}1^+$) in SDH. Scale, 20 μm. (**b**) Prevention of spinal nerve ligation (SNL)-induced heat hyperalgesia by intra-spinal pretreatment of *Arrb2*-lentivirus (*Arrb2*-LV) via microinjections, given one week before SNL. *P<0.05, two-way ANOVA, n = 5 mice/group. (**c**) Reversal of SNL-induced heat hyperalgesia by intra-spinal post-treatment of *Arrb2*-LV, given one week after SNL. *P<0.05, two-way ANOVA, followed by post-hoc Bonferroni test. n = 9 mice/group. All data are expressed as mean ± S.E.M.

Full length western blots of Figure 3a

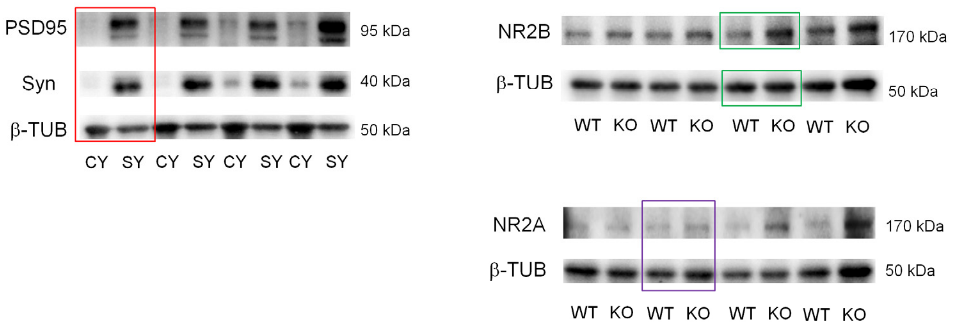

Full length western blots of Figure 3c

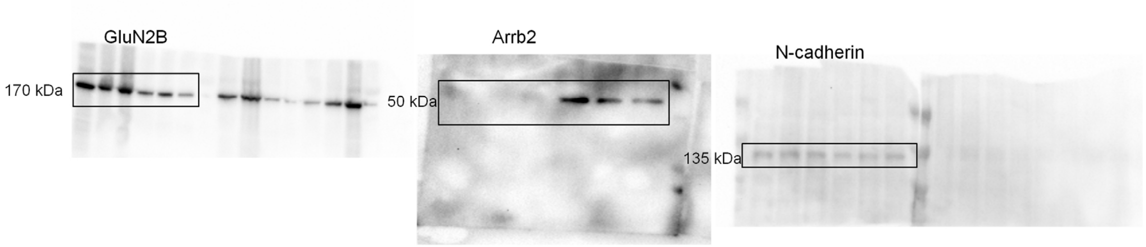

Full length western blots of Figure 3e

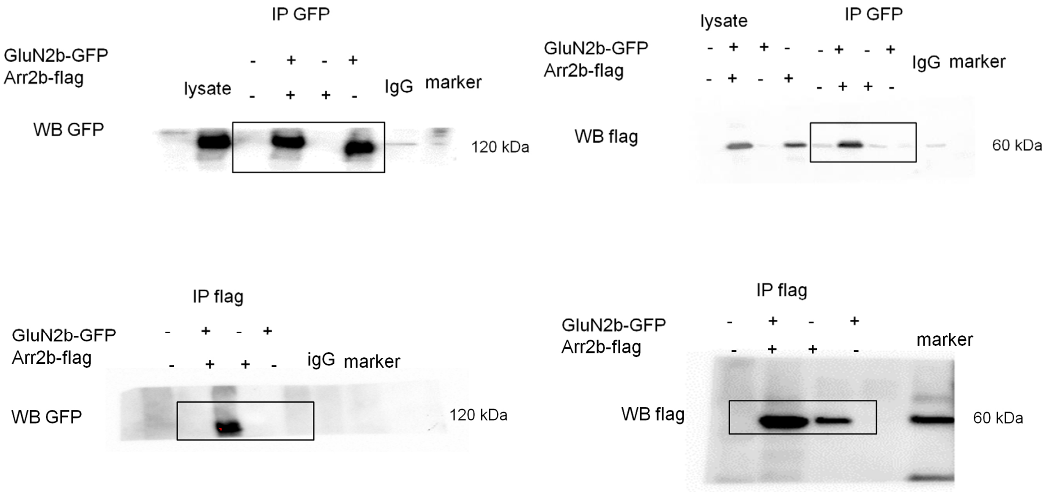

Supplementary Figure 8a. Full size western blots of Figure 3a, 3c and 3e, obtained from the respective boxes. Cy, cytosol; Sy, synaptosome; NR2A, GluN2A; NR2B, GluN2B.

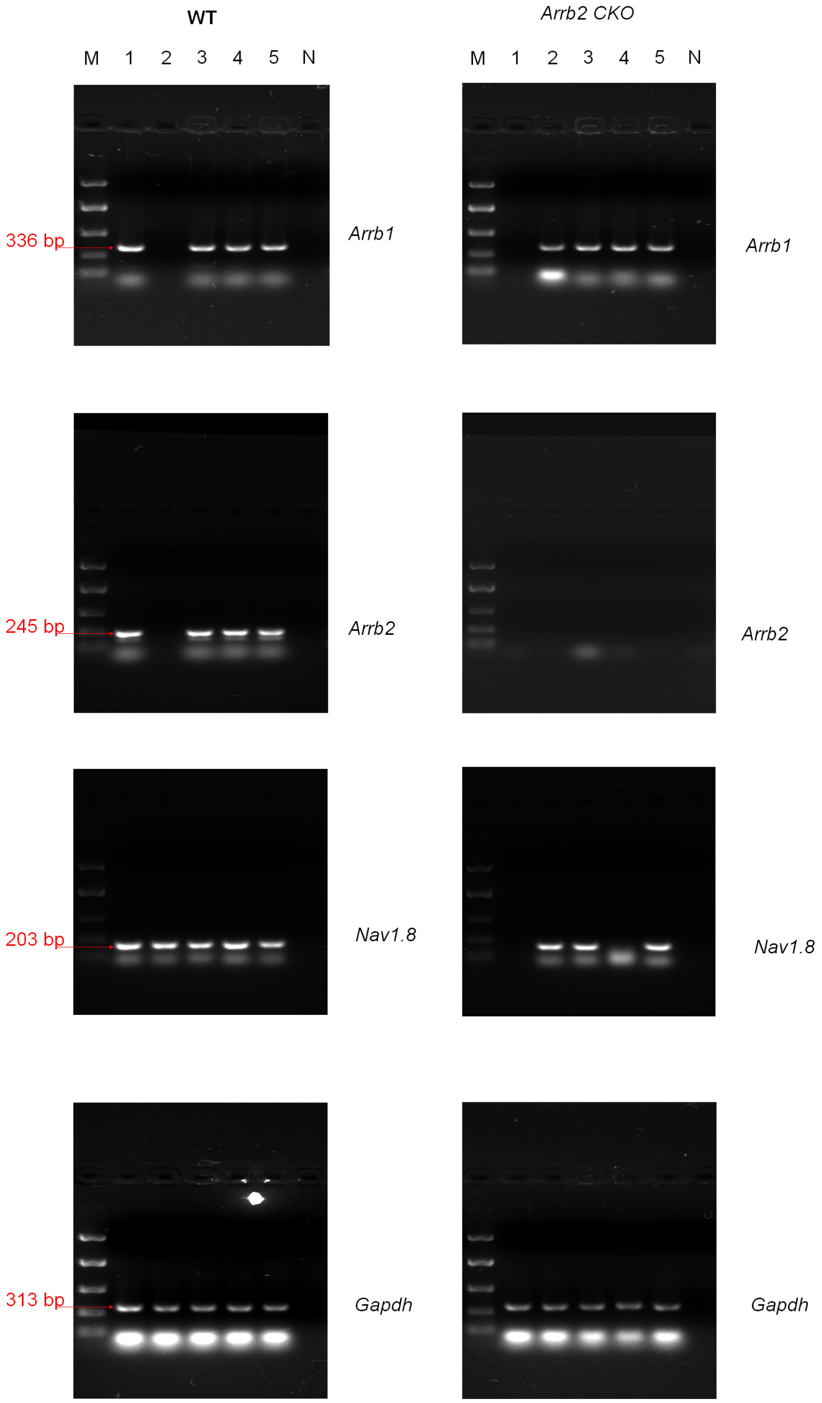

Supplementary Figure 8b. Full size gels of Figure 7a.

Full length western blots of Supplementary Figure 2a

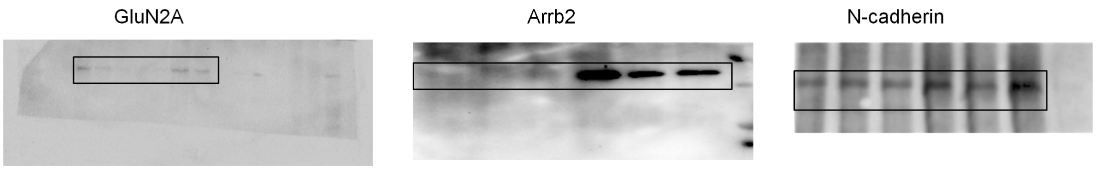

Full length western blots of Supplementary Figure 2c

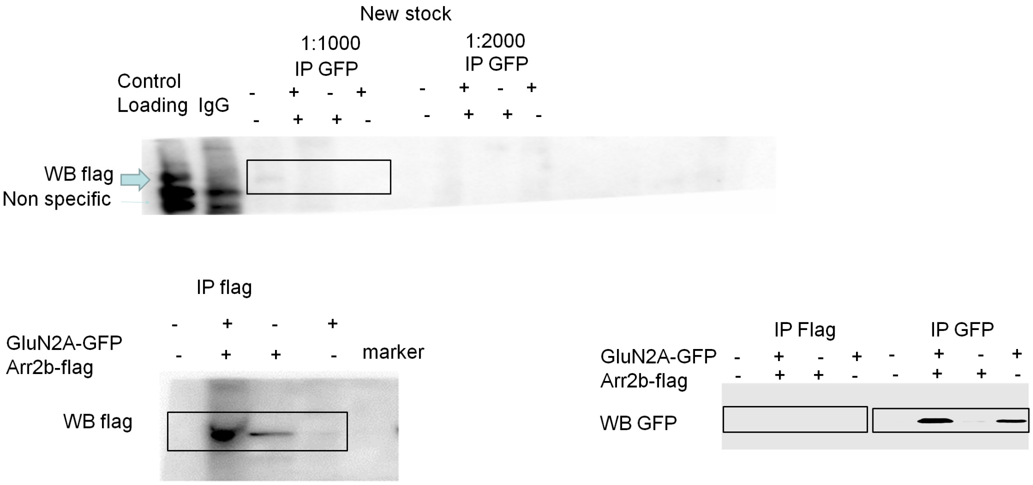

Supplementary Figure 8c. Full size western blots of Supplementary Figure 2a and 2c.

Supplementary Table 1. Sample sizes and number of animals used in this study.

Experiment	Sample size	Number of Groups	Number of samples	Number of mice
**1. In vivo**				
1.1 Behavioral test	n = 4-14 mice	53	282 mice	282
1.2 WB	n = 4 mice	2	8 mice	8
1.3 Immunohistochemistry	n = 4 mice	2	8 mice	8
1.4 ISH	n = 4 mice	2	8 mice	8
1.5 RT-PCR				
1.5.1 Dorsal horn	n = 5 mice	3	15 mice	15
1.5.2 Single cell	n = 5 neurons	2	10 neurons	4
**2. Ex vivo**				
2.1 Patch-clamp recording	n = 5-21 neurons	32	243 neurons	115
**3. In vitro**				
3.1 Hela cell culture	n = 3 cultures	12	36 cultures	0
		Total number of mice		**440**

Supplementary Table 2. List of primers for single-cell PCR.

Target gene (Product length) [a]	Outer primers	Inner primers	Genbank No.
Arrb1 (709 bp, 336 bp)	AGGAGAGAGAAAGAGACAGAGG<br>GAGGAACAGAAACGGAGATGAG	CTCTTTGGGCCTCAGTTTCT<br>AGGTTGTTCCCACACTTCTC	NM_177231
Arrb2 (332 bp, 245 bp)	GAACTCTGTGCGGCTTATCA<br>CCTTGCAGAACGTGGAACTA	TGAAACCACACGCCACTT<br>TAGGAGACACCTGGTCATCTT	NM_001271358
Nav1.8 (316 bp, 203 bp)	CATGACAGAGGAGCAGAAGAAG<br>CCAGCCGTTGGTGAAGTAATA	CTTTGAATAAGTACCAGGGCTTC<br>GAACATCTTCATCACACACTCG	NM_001205321
Gapdh (367 bp, 313 bp)	AGCCTCGTCCCGTAGACAAAA<br>TTTTGGCTCCACCCCTTCA	TGAAGGTCGGTGTGAACGAATT<br>GCTTTCTCCATGGTGGTGAAGA	XM_001473623

[a] (n, n) indicates product size obtained from outer and inner primers, respectively.
